# Supplementary material for: Impact of Ginkgo biloba drug interactions on bleeding risk and coagulation profiles: A comprehensive analysis
Source: PLoS One. 2025 Apr 8;20(4):e0321804. doi: 10.1371/journal.pone.0321804 (PMC11991284; doi:10.1371/journal.pone.0321804)
Supplement: Attachment — Submitted filename: PONE-D-24-33374_Reviewed.pdf [file pone.0321804.s005.pdf]

PLOS ONE

Impact of Ginkgo biloba drug interactions on bleeding risk and coagulation profiles: A comprehensive analysis

--Manuscript Draft--

Manuscript Number:	PONE-D-24-33374R1
Article Type:	Research Article
Full Title:	Impact of Ginkgo biloba drug interactions on bleeding risk and coagulation profiles: A comprehensive analysis
Short Title:	Impact of Ginkgo Biloba Interactions on Bleeding Risk and Coagulation
Corresponding Author:	Phuong Nguyen Thi Thu, MD, PhD<br>Hai Phong University of Medicine and Pharmacy: Hai Phong Medical University<br>Haiphong, VIET NAM
Keywords:	ginkgo Biloba; herb-drug interactions; bleeding Risk; coagulation Tests; Anticoagulants
Abstract:	This retrospective observational study was conducted to investigate the prevalence and implications of drug interactions involving Ginkgo biloba extract on bleeding risk and coagulation profiles. Among the 2,647 prescriptions that met the inclusion criteria, 342 exhibited drug interactions with a prevalence rate of 12.94%. Notably, Ginkgo biloba extract primarily interacts with antiplatelets, anticoagulants, and nonsteroidal anti-inflammatory drugs, with Clopidogrel and Aspirin exhibiting the highest prevalence rates of 2.61% each. However, the interactions with anticoagulants, particularly direct oral anticoagulants and acenocoumarol, were not significant in our analysis. Omeprazole was a frequently interacting drug (2.34%) of mild severity. Among the 747 patients analyzed for bleeding disorders, 31 (4.15%) exhibited bleeding symptoms. Correlation analysis indicated a strong association between clinical bleeding and abnormal coagulation results (OR, 1.75; p < 0.001). Moreover, significant correlations were found between Ginkgo biloba extract drug interactions and the bleeding risk (OR: 1.08, p < 0.001) and abnormal coagulation (OR: 1.49, p < 0.001). The severity of Ginkgo biloba extract drug interactions did not correlate with bleeding risk (OR: 1.01, p = 0.767) but influenced abnormal coagulation test results (OR: 0.813, p = 0.019). Specific medications, including clopidogrel, aspirin, celecoxib, loxoprofen, nifedipine, and omeprazole, were significantly associated with the risk of bleeding and abnormal coagulation (p < 0.05). Interactions with ticagrelor, etoricoxib, insulin, omeprazole, and domperidone were associated with abnormal coagulation tests without affecting the reported bleeding. These findings emphasize the importance of considering Ginkgo biloba extract drug interactions in bleeding risk assessment and coagulation management.
Order of Authors:	Ngo Thi Quynh Mai, PhD
	Nguyen Viet Hieu, MS
	Tran Thi Ngan, PhD
	Tran Van Anh, MS
	Pham Van Linh, MD, PhD
	Phuong Nguyen Thi Thu, MD, PhD
Response to Reviewers:	Response to reviewers:<br>The authors did well in the conceptualization of the study. However, the manuscript was not properly written. The result and discussion sessions need a major revision. There was no Figure 1 in the result session. I have highlighted areas where revision should be made.<br>Thank you for your feedback regarding our manuscript. We appreciate your recognition of the conceptualization of the study.<br>We acknowledge your comments about the need for major revisions in the results and discussion sections; we have taken the necessary steps to enhance clarity and coherence throughout these areas.

Powered by Editorial Manager® and ProduXion Manager® from Aries Systems Corporation

	Regarding Figure 1, we understand that it was submitted separately in accordance with the guidelines of PLOS ONE. However, we will resubmit Figure 1 along with the revised manuscript to ensure it is readily available for review and aligns with the manuscript content.<br>We believe that the revisions made have significantly improved the manuscript, and we appreciate your valuable guidance in this process. Thank you for your consideration.<br>Line 23-40:<br>1. start the abstract with a little introduction on Gingo biloba<br>2. there is no methodology in this abstract. What was the selection criteria?<br>kindly include statistical analysis that was used.<br>Thank you for your insightful feedback regarding our abstract. We have made the recommended changes to enhance its clarity and comprehensiveness.<br>1.We have added an introductory statement about Ginkgo biloba at the beginning of the abstract to provide context for the study. This introduction helps establish the significance of Ginkgo biloba in relation to the research conducted.<br>2.We acknowledge the lack of methodological details in the original abstract. In response to your comments, we have included the selection criteria used for participant inclusion in the study. Additionally, we have specified the statistical analysis methods employed to assess the data.<br>The following paragraph has been added to our abstract to address these points:<br>"Our analysis utilized data from patients admitted to Hai Phong International Hospital between January 2022 and December 2023. Inclusion criteria consisted of patients aged 18 years and above, those prescribed Ginkgo biloba extract alone or in combination with other medications, and the availability of complete medical records, including medication history, laboratory tests, and clinical outcomes"<br>3. Add keywords<br>The following keywaords has been added to our manuscript:<br>Ginkgo biloba; drug interactions; bleeding risk; anticoagulants; coagulation profiles<br>Line 47 -48: reference???<br>In response to your inquiry about the reference for lines 47-48, we have added the appropriate citation to support the statements made in that section.<br>Line 51: repetition: G. biloba leaf extract (EGb) is widely recognized as one of the most popular health supplements globally due to its benefits for mental focus. EGb 761, the standardized EGb, is commonly used in studies investigating its effects. Research has indicated that EGb influences several neurotransmitter pathways and brain structures, particularly those observed in animal studies<br>In response to your feedback, we have revised this section to eliminate redundant phrases.<br>Line 73: Even though EGb is commonly used, it has risks, especially in terms of its potential interactions with prescription medications. These interactions can lead to issues like bleeding disorders and abnormal coagulation profiles. Consequently, caution is advised when administering EGb to patients with bleeding disorders or those who are taking NSAIDs, antiplatelet drugs, or anticoagulants.<br>In response to your feedback, we have revised this section based on your suggestions<br>Line 81: Recently, several case reports have suggested a possible connection between hemorrhagic complications and the use of EGb preparations. In response, a trial was carried out to evaluate the effects of the Ginkgo biloba special extract, EGb 761, on hemostatic parameters. The study found that, out of the 29 coagulation and bleeding parameters examined, EGb 761 did not show any evidence of inhibiting blood coagulation or platelet aggregation.<br>In response to your feedback, we have revised this section based on your suggestions<br>Line 114: Study design<br>In response to your feedback, we have revised this section based on your suggestions as following:<br>Patients aged 18 years and older<br>Patients prescribed EGb either alone or alongside other prescription medications<br>Availability of complete medical records, including medication history, laboratory tests, and clinical outcomes.<br>This criterion was specifically applied to examine the impact of EGb interactions on bleeding occurrences or abnormal coagulation tests.<br>Line 176-194: kindly summarize. some of the information should be moved to the discussion session<br>Discussion was poorly written.<br>Kindly rewrite this section.

Powered by Editorial Manager® and ProduXion Manager® from Aries Systems Corporation

	1. the result was not properly discussed. kindly discuss the result . move some of the information in the results section to this section<br>2. authors should check for grammar. use the right tenses.<br><br>Thank you for your constructive feedback regarding the discussion section of our manuscript. We appreciate your attention to detail and the suggestions for improvement.<br>In response to your comments, we have rewritten the discussion to provide a more thorough analysis of the results. We have moved relevant information from the results section to ensure that findings are properly contextualized and discussed in detail. Additionally, we have carefully reviewed the discussion for grammatical accuracy and proper use of tenses, making necessary adjustments to enhance clarity and readability.<br>We believe that the revisions have significantly strengthened the discussion section, and we appreciate your guidance in helping us improve the overall quality of the manuscript. Thank you for your valuable input.
**Additional Information:**	
**Question**	**Response**
**Financial Disclosure**<br><br>Enter a financial disclosure statement that describes the sources of funding for the work included in this submission. Review the submission guidelines for detailed requirements. View published research articles from *PLOS ONE* for specific examples.<br><br>This statement is required for submission and **will appear in the published article** if the submission is accepted. Please make sure it is accurate.<br><br>**Funded studies**<br>Enter a statement with the following details:<br>• Initials of the authors who received each award<br>• Grant numbers awarded to each author<br>• The full name of each funder<br>• URL of each funder website<br>• Did the sponsors or funders play any role in the study design, data collection and analysis, decision to publish, or preparation of the manuscript?<br><br>Did you receive funding for this work?	The author(s) received no specific funding for this work.
**Competing Interests**<br><br>Use the instructions below to enter a competing interest statement for this	The authors have declared that no competing interests exist

submission. On behalf of all authors, disclose any competing interests that could be perceived to bias this work—acknowledging all financial support and any other relevant financial or non-financial competing interests.<br><br>This statement is required for submission and **will appear in the published article** if the submission is accepted. Please make sure it is accurate and that any funding sources listed in your Funding Information later in the submission form are also declared in your Financial Disclosure statement.<br><br>View published research articles from *PLOS ONE* for specific examples.<br><br>**NO authors have competing interests**<br><br>Enter: *The authors have declared that no competing interests exist.*<br><br>**Authors with competing interests**<br><br>Enter competing interest details beginning with this statement:<br><br>*I have read the journal's policy and the authors of this manuscript have the following competing interests: [insert competing interests here]*<br><br>* typeset	
**Ethics Statement**<br><br>Enter an ethics statement for this submission. This statement is required if the study involved:<br><br>• Human participants<br>• Human specimens or tissue<br>• Vertebrate animals or cephalopods<br>• Vertebrate embryos or tissues<br>• Field research<br><br>Write "N/A" if the submission does not	This study adhered to the ethical principles outlined in the Declaration of Helsinki. Patient confidentiality and privacy were maintained throughout the data collection and analysis. Institutional Review Board (IRB.23.128) approval was obtained before the commencement of data collection and analysis. The study was conducted utilizing routine hospital data, and in accordance with the Board regulations, the acquisition of informed consent from individual participants was not deemed necessary.

Powered by Editorial Manager® and ProduXion Manager® from Aries Systems Corporation

require an ethics statement.<br><br>General guidance is provided below. Consult the submission guidelines for detailed instructions. **Make sure that all information entered here is included in the Methods section of the manuscript.**<br><br>**Format for specific study types**<br><br>**Human Subject Research (involving human participants and/or tissue)**<br>• Give the name of the institutional review board or ethics committee that approved the study<br>• Include the approval number and/or a statement indicating approval of this research<br>• Indicate the form of consent obtained (written/oral) or the reason that consent was not obtained (e.g. the data were analyzed anonymously)<br><br>**Animal Research (involving vertebrate animals, embryos or tissues)**<br>• Provide the name of the Institutional Animal Care and Use Committee (IACUC) or other relevant ethics board that reviewed the study protocol, and indicate whether they approved this research or granted a formal waiver of ethical approval<br>• Include an approval number if one was obtained<br>• If the study involved *non-human primates*, add *additional details* about animal welfare and steps taken to ameliorate suffering<br>• If anesthesia, euthanasia, or any kind of animal sacrifice is part of the study, include briefly which substances and/or methods were applied<br><br>**Field Research**<br><br>Include the following details if this study involves the collection of plant, animal, or other materials from a natural setting:<br>• Field permit number<br>• Name of the institution or relevant body that granted permission	
**Data Availability**	Yes - all data are fully available without restriction

Powered by Editorial Manager® and ProduXion Manager® from Aries Systems Corporation

Authors are required to make all data underlying the findings described fully available, without restriction, and from the time of publication. PLOS allows rare exceptions to address legal and ethical concerns. See the PLOS Data Policy and FAQ for detailed information.<br><br>A Data Availability Statement describing where the data can be found is required at submission. Your answers to this question constitute the Data Availability Statement and **will be published in the article**, if accepted.<br><br>**Important:** Stating 'data available on request from the author' is not sufficient. If your data are only available upon request, select 'No' for the first question and explain your exceptional situation in the text box.<br><br>Do the authors confirm that all data underlying the findings described in their manuscript are fully available without restriction?	
**Describe where the data may be found in full sentences. If you are copying our sample text, replace any instances of XXX with the appropriate details.**<br><br>• If the data are **held or will be held in a public repository**, include URLs, accession numbers or DOIs. If this information will only be available after acceptance, indicate this by ticking the box below. For example: *All XXX files are available from the XXX database (accession number(s) XXX, XXX.).*<br>• If the data are all contained **within the manuscript and/or Supporting Information files**, enter the following: *All relevant data are within the manuscript and its Supporting Information files.*<br>• If neither of these applies but you are able to provide **details of access elsewhere**, with or without limitations, please do so. For example:	All relevant data are within the manuscript and its Supporting Information files.

Data cannot be shared publicly because of [XXX]. Data are available from the XXX Institutional Data Access / Ethics Committee (contact via XXX) for researchers who meet the criteria for access to confidential data.<br><br>*The data underlying the results presented in the study are available from (include the name of the third party and contact information or URL).*<br>• This text is appropriate if the data are owned by a third party and authors do not have permission to share the data.<br><br>* typeset	
Additional data availability information:	Tick here if the URLs/accession numbers/DOIs will be available only after acceptance of the manuscript for publication so that we can ensure their inclusion before publication.

Manuscript
Click here to access/download;Manuscript;Manuscripts_DDI_clean.doc

Impact of Ginkgo biloba drug interactions on bleeding risk and coagulation profiles: A comprehensive analysis

Short title: Impact of Ginkgo Biloba Interactions on Bleeding Risk and Coagulation

Ngo Thi Quynh Mai[1], Nguyen Viet Hieu[1], Tran Thi Ngan[1,2], Tran Van Anh[1,2], Pham Van Linh[3], Nguyen Thi Thu Phuong[1,2]

[1] Faculty of Pharmacy, Hai Phong University of Medicine and Pharmacy, Hai Phong 180000, Vietnam

[2] Department of Pharmacy, Hai Phong International Hospital, Hai Phong 180000, Vietnam

[3] Department of Pathology and Immunology, Hai Phong University of Medicine and Pharmacy, Hai Phong 180000, Vietnam

* Corresponding author:

Email: nttphuong@hpmu.edu.vn (NTTP)

Abstract

This retrospective observational study was conducted to investigate the prevalence and implications of drug interactions involving *Ginkgo biloba* extract on bleeding risk and coagulation profiles. Our analysis utilized data from patients admitted to Hai Phong International Hospital between January 2022 and December 2023. Inclusion criteria consisted of patients aged 18 years and above, those prescribed *Ginkgo biloba* extract alone or in combination with other medications, and the availability of complete medical records, including medication history, laboratory tests, and clinical outcomes. Among the 2,647 prescriptions that met the inclusion criteria, 342 exhibited drug interactions with a prevalence rate of 12.94%. *Ginkgo biloba* extract primarily interacted with antiplatelets, anticoagulants, and nonsteroidal anti-inflammatory drugs, with Clopidogrel and Aspirin exhibiting the highest prevalence rates of 2.61% each. However, the interactions with anticoagulants, particularly direct oral anticoagulants and acenocoumarol, were not significant. Omeprazole was a frequently interacting drug (2.34%) of mild severity. Among the 747 patients analyzed for bleeding disorders, 31 (4.15%) exhibited bleeding symptoms. Correlation analysis indicated a strong association between clinical bleeding and abnormal coagulation results (OR, 1.75; $p < 0.001$). Moreover, significant correlations were found between *Ginkgo biloba* extract drug interactions and the bleeding risk (OR: 1.08, $p < 0.001$) and abnormal coagulation (OR: 1.49, $p < 0.001$). The severity of *Ginkgo biloba* extract drug interactions did not correlate with bleeding risk (OR: 1.01, $p = 0.767$) but influenced abnormal coagulation test results (OR: 0.813, $p = 0.019$). Specific medications, including clopidogrel, aspirin, celecoxib, loxoprofen, nifedipine, and omeprazole, were significantly associated with the risk of bleeding and abnormal coagulation ($p < 0.05$). Interactions with

ticagrelor, etoricoxib, insulin, omeprazole, and domperidone were associated with abnormal coagulation tests without affecting the reported bleeding. These findings emphasize the importance of considering *Ginkgo biloba* extract drug interactions in bleeding risk assessment and coagulation management.

Keywords: Ginkgo biloba; drug interactions; bleeding risk; anticoagulants; coagulation profiles

Introduction

Ginkgo biloba extract, a herbal supplement derived from the leaves of the species *Ginkgo biloba (G. biloba)*, has gained popularity worldwide owing to its perceived health benefits, including cognitive enhancement and peripheral circulation improvement (1, 2). *G. biloba* leaves contain two primary active ingredients: terpene lactones (including ginkgolides and diterpenes) and Ginkgo flavone glycosides (containing ginkgetin, bilobetin, and sciadopitysin), which are present in varying concentrations (3). Known for its positive effects on mental focus, *G. biloba* leaf extract (EGb) is recognized as one of the most widely used health supplements. The standardized version, EGb 761, is frequently employed in research exploring its effects. Studies have shown that EGb affects various neurotransmitter systems and brain structures, particularly evident in animal research (4, 5). EGb 761 reduces stress-induced corticosterone hypersecretion in rats by decreasing the number of peripheral adrenal benzodiazepine receptors (6). However, the efficacy of EGb 761 in treating dementia has been a topic of debate, with conflicting findings from various studies. A 52-week randomized, double-blind, placebo-controlled trial involving 309 patients suggested that EGb 761 was safe and, albeit modestly, appeared to stabilize and improve cognitive performance and social functioning in patients with dementia for 6 months to 1 year (7). Systematic reviews conducted in 2009 and 2018, comprising 36 and 38 trials, respectively,

highlighted the safety of EGb but did not find sufficient evidence to support its clinical benefit in patients with cognitive impairment and dementia (8). Conversely, a 2015 systematic review of nine trials suggested that EGb 761 at a dose of 240 mg/day could slow the decline in cognition, function, behavior, and overall global change at 22–26 weeks, particularly in patients with neuropsychiatric symptoms (9). Overall, EGb is regarded as safe and generally well-tolerated, with a maximum recommended dose of 240 mg/day (10). Mild side effects may include headache, heart palpitations, gastrointestinal issues, constipation, and allergic skin reactions (3). Although a systematic review and meta-analysis found no significant effect of EGb on prothrombin time, activated partial thromboplastin time, or platelet aggregation, several case reports have noted a temporal link between EGb use and bleeding events, including severe intracranial bleeding (11, 12).

Although is commonly used, it carries certain risks, particularly regarding its potential interactions with prescription medications. Such interactions may result in complications like bleeding disorders and abnormal coagulation profiles (13). Therefore, it is important to exercise caution when administering EGb to patients with bleeding disorders or those using NSAIDs, antiplatelet drugs, or anticoagulants (14)(15).

Recently, several case reports have indicated a possible association between hemorrhagic complications and the use of EGb preparations. In light of these concerns, a trial was conducted to assess the effects of the *G. biloba* special extract, EGb 761, on hemostatic parameters. The results revealed that none of the 29 coagulation and bleeding parameters evaluated showed any evidence of EGb 761 inhibiting blood coagulation or platelet aggregation. Furthermore, the study did not reveal any evidence to substantiate the causal relationship between EGb administration of EGb 761 and hemorrhagic complications (16). However, the concomitant use of EGb with

prescription drugs has raised concerns among healthcare professionals owing to the potential for pharmacokinetic and pharmacodynamic interactions. These interactions can alter the efficacy and safety profiles of EGb and co-administered medications, posing significant challenges in clinical management.

Through a retrospective observational study design, we sought to clarify the frequency and severity of drug interactions involving EGb, specifically in relation to bleeding risk and abnormalities in coagulation profiles in a real-world clinical context. The results of this study could inform clinical decision-making, improve patient care, and enhance medication safety for individuals undergoing EGb therapy in combination with prescription medications.

Materials and methods

Study design

This retrospective observational study analyzed data collected from the medical records of patients admitted to Hai Phong International Hospital (Hai Phong, Vietnam) between January 2022 and December 2023. Data were accessed for study purposes on February 15, 2024.

Inclusion criteria:

- Patients aged 18 years and older
- Patients prescribed EGb either alone or in combination other prescription medications
- Availability of complete medical records, including medication history, laboratory tests, and clinical outcomes.

This criterion was specifically applied to assess the impact of EGb interactions on bleeding occurrences or abnormal coagulation tests.

Exclusion criteria:

- Patients with incomplete medical records
- Patients with a history of bleeding disorders unrelated to medication use
- Patients prescribed herbal supplements other than EGb
- Patients with missing demographic or clinical data

Data extraction:

A structured data extraction form was used to collect the following information:

- Patient demographics (age, gender, weight, body mass index [BMI])
- Medication history, including EGb and other prescription drugs
- Laboratory tests, including activated partial thromboplastin time (APTT), prothrombin time (PT), and fibrinogen levels
- Clinical outcomes such as bleeding events and diagnosis of bleeding disorders
- Severity classification of drug interactions based on established criteria

Identification of drug interactions:

Drug interactions involving EGb were identified using reputable drug interaction databases such as UpToDate/Micromedex/Drugs.com. Interactions were classified based on severity (mild, moderate, or severe) and documented adverse effects, particularly bleeding disorders and abnormal coagulation profiles.

Statistical analyses

Descriptive statistics were used to summarize the patient demographics, medication profiles, and clinical outcomes. The prevalence of drug interactions with EGb was calculated as the proportion of patients with documented interactions. Chi-square tests were used to assess associations between categorical variables, whereas t-tests ~~tests~~ were used for continuous variables. Logistic regression analysis was performed to identify the factors associated with bleeding events and abnormal coagulation test results. Statistical significance was determined at $p < 0.05$. The logistic regression model was built using the R statistical software, version 3.2.4 (A Language and Environment for Statistical Computing, Vienna, Austria) (17).

Ethical considerations

This study adhered to the ethical principles outlined in the Declaration of Helsinki. Patient confidentiality and privacy were maintained throughout the data collection and analysis. Institutional Review Board (IRB.23.128) approval was obtained before the commencement of data collection and analysis. All patients provided verbal consent for the use of their data in research at the time of their initial treatment. This consent included an explanation of the potential use of their anonymized data in future research studies.

Results

To calculate the frequency and rate of drug interactions between EGb and other prescription drugs, we selected 2,647 prescriptions that met the inclusion and exclusion criteria. Among these, 342 prescriptions exhibited drug interactions, resulting in a prevalence of 12.94%. The study diagram is presented in Fig 1.

Figure 1. Study diagram

The interaction between EGb and conventional drugs is presented in Table 1. EGb interacts predominantly with antiplatelet drugs, anticoagulants, and NSAIDs. Interactions with antiplatelet drugs manifested the highest prevalence rate, coupled with an average severity level. Specifically, interactions with clopidogrel and aspirin were documented in 69 prescriptions, representing a rate of 2.61%.

Furthermore, interactions between EGb and anticoagulants have also garnered significant attention owing to their notable prevalence rates. Noteworthy interactions were observed with direct oral anticoagulants (DOACs), documented in 20 prescriptions (18%), and with acenocoumarol, noted in 13 prescriptions (0.49%). According to UpToDate/Micromedex, interactions between EGb and agents with antiplatelet properties/anticoagulants may potentiate adverse or toxic effects, including bleeding.

Within the spectrum of interactions between EGb and other drugs, omeprazole emerged at a notable frequency, as documented in 62 prescriptions, constituting 2.34% of the observed interactions. Despite the relatively high rate, the severity of this interaction pair was considered mild.

Out of 2,647 patients prescribed EGb preparations, 747 were eligible for Part 2 of the study, focusing on the relationship between EGb drug interactions and bleeding disorders. Among these, 489 patients without drug interactions formed the control group, while 258 patients had drug interactions. The characteristics of these patients, including weight, age, and sex, are detailed in Table 2. Notably, 31 patients (4.15%) were diagnosed with bleeding disorders or symptoms. The clinical characteristics and drug interactions of this subset were analyzed,

revealing a mean activated partial thromboplastin time (APTT) of 57.25 ± 9.34 seconds, a prothrombin time (PT) of 110.71 ± 26.18%, and a fibrinogen level of 3.56 ± 1.25 g/L.

To clarify the relationship between EGb drug interactions and bleeding disorders, a control group of 484 patients without drug interactions was established, alongside 258 patients who had interactions with other medications. Among these patients, the interaction pairs were distributed as follows: 185 patients had one interaction (61.24%), 64 had two (24.81%), seven had three (2.71%), and two had four (0.77%). Of the 258 patients with drug interactions, 226 (87.6%) experienced moderate severity interactions, while 32 (12.4%) had mild severity interactions, as outlined in Table 2.

Table 1: Interaction between EGb and conventional drugs (n=2,647)

Drug class	Prescribed drug	Number of DDIs	Frequency (%)	Severity	Mechanism	Consequence	Reference
Agents with antiplatelet properties	Clopidogrel	69	2.61	Moderate	Many herbal products have been shown to inhibit platelet function, prolong bleeding time, or contribute to bleeding events in people. Concomitant use of these herbal products with drugs that have a similar pharmacologic potential may increase the risk of bleeding.	Enhance the adverse/toxic effects of agents with antiplatelet properties. Bleeding may occur	UpToDate
	Aspirin	69	2.61	Moderate			
	Ticagrelor	2	0.08	Moderate			
Anticoagulants	DOAC (enoxaban/ rivaroxaban)	18	0.68	Moderate	Many herbal products possess the ability to cause or potentiate bleeding (inhibit clotting/coagulation or primary hemostasis) by one of several mechanisms. They may inhibit platelet aggregation, inhibit cyclooxygenase activity, interfere with one or more components of the coagulation cascade, or increase bleeding risk by another mechanism. The concomitant use of such herbs with other herbs or drugs possessing a similar pharmacologic potential may increase the risk of bleeding.	Enhance the adverse/toxic effects of anticoagulants. Bleeding may occur.	UpToDate
	Acenocoumarol	13	0.49	Moderate			

Drug class	Prescribed drug	Number of DDIs	Frequency (%)	Severity	Mechanism	Consequence	Reference
Nonsteroidal anti-inflammatory drugs (NSAIDs)	Etoricoxib	20	0.76	Moderate	Many herbal products have been shown to inhibit platelet function, prolong bleeding time, or contribute to bleeding events in people. Concomitant use of these herbal products with drugs that have a similar pharmacologic potential (such as NSAIDs) may increase the risk of bleeding.	Enhance the adverse/toxic effects of NSAIDs. Bleeding may occur	UpToDate
	Celecoxib	14	0.53	Moderate			
	Meloxicam	10	0.38	Moderate			
	Loxoprofen	2	0.08	Moderate			
Other	Nifedipine	24	0.91	Moderate	Inhibition of cytochrome P450 3A4 by *G. biloba*	Concurrent use of *G. biloba* and nifedipine may result in an increased risk of nifedipine side effects.	UpToDate
	Insulin	22	0.83	Moderate	*G. biloba* may increase pancreatic beta-cell function	Concurrent use of *G. biloba* and insulin may result in altered insulin effectiveness.	Micromedex
	Omeprazol	62	2.34	Minor	The mechanism of this potential interaction is	Decrease the serum	Microme

Drug class	Prescribed drug	Number of DDIs	Frequency (%)	Severity	Mechanism	Consequence	Reference
	e				unknown. While *G. biloba*-mediated induction of CYP2C19 has been proposed as a potential mechanism, the large effect seen in CYP2C19 poor metabolizers combined with a lack of effects seen with voriconazole and diazepam (substrates of CYP2C19) in other studies [3,4] makes the role of CYP2C19 induction in this interaction questionable. Additionally, because natural products have variable constituents, it is unknown if a particular formulation or dose is more susceptible to this interaction.	concentration of omeprazole	dex
	Domperidone	17	0.64	Minor	Inhibition of CYP3A4-mediated domperidone metabolism	Concurrent use of domperidone and *G. biloba* may result in increased domperidone exposure and an	Micromedex

Drug class	Prescribed drug	Number of DDIs	Frequency (%)	Severity	Mechanism	Consequence	Reference
						increased risk of QT prolongation.	
Total		342	12.94				

DDIs: Drug-drug interactions; DOAC: Direct oral anticoagulant; NSAIDs: Nonsteroidal anti-inflammatory drugs.

Table 2: Clinical characteristics and drug interactions of the patient group to evaluate the relationship between DDI of EGb - bleeding/coagulopathy (n=747)

	**No DDI (n=489)**	**DDI (n=258)**	**Total (n=747)**
Weight (kg, mean±SD)	57.14±9.44	57.46±9.15	57.25±9.34
Age (years, mean±SD)	54.26±15.79	63.08±13.93	57.31±15.74
Sex (Ref: female) (n,%)	331 (67.69%)	172 (66.67%)	503 (67.34%)
BMI (mean±SD)	22.62±2.81	23.09±2.78	22.78±2.81
Symptom of bleeding (Ref: yes) n,%)	8(1.64%)	23(8.91%)	31(4.15%)
APTT (s) (mean±SD)	33.3±4.56	34.11±5.45	33.78±5.12
PT (%, mean±SD)	102.2±17.31	126.83±31.92	110.71±26.18
Fibrinogen (g/l, mean±SD)	3.19±0.7	5.24±1.78	3.55±1.25
Abnormal coagulant test (n,%)	127 (25.97%)	169 (65.5%)	296 (39.63%)
Number of DDIs (n,%)			
One interaction pair		185 (71.71%)	185 (24.77%)
Two interaction pairs		64 (24.81%)	64 (8.57%)
Three interaction pairs		7 (2.71%)	7 (0.94%)
Four interaction pairs		2 (0.78%)	2 (0.27%)
Severity of DDI (n=258)			
Moderate		32 (12.4%)	32 (4.28%)
Minor		226 (87.6%)	715 (95.72%)

DDIs: Drug-Drug Interactions; SD: Standard Deviation; BMI: Body Mass Index; APTT: Activated Partial Thromboplastin Time; PT: Prothrombin Time

Investigate how clinical variables and drug interaction characteristics are associated with the incidence of clinically documented bleeding and abnormalities in coagulation test results among

747 patients. The results indicate that weight and BMI did not significantly impact the occurrence of recorded bleeding or abnormalities in coagulation test results ($p > 0.05$, as shown in Table 3). Although sex did not demonstrate a substantial association with the occurrence of bleeding, it exhibited a slight correlation with abnormalities in coagulation test results, with an odds ratio (OR) of 1.07 (95% confidence interval (6): 0.99; 1.15) ($p = 0.071$).

The study found a significant association between clinical bleeding cases and abnormal coagulation results, with an odds ratio (OR) of 1.75 (95% CI: 1.48; 2.08), indicating a strong correlation ($p < 0.001$). Each coagulation test APTT, PT, and fibrinogen levels demonstrated a strong relationship with the occurrence of clinical bleeding.

Furthermore, the study revealed a noteworthy link between drug interactions involving EGb and prescription medications and the occurrence of bleeding and abnormal coagulation test results. The odds ratios for these associations were found to be 1.08 (95% CI: 1.04; 1.11) and 1.49 (95% CI: 1.38; 1.58), respectively, implying a significant correlation ($p < 0.001$). These findings underscore the clinical relevance of drug interactions, particularly those involving EGb, in the context of bleeding risk and abnormal coagulation profiles.

The study found no correlation between the severity of drug interactions involving EGb and the occurrence of bleeding, with an odds ratio (OR) of 1.01 (95% CI: 0.94; 1.08) and a p-value of 0.767, indicating no significant association. However, a significant effect was observed on abnormalities in coagulation test results, with an OR of 0.813 (95% CI: 0.68; 0.96) and a p-value of 0.019.

When examining the relationship between drug interactions involving EGb and specific medications and the occurrence of bleeding, our analysis revealed that several drugs exhibited

statistical significance with a p-value of less than 0.05. These drugs include clopidogrel, aspirin, celecoxib, loxoprofen, nifedipine, and omeprazole.

The study identified statistically significant correlations between drug interactions involving EGb and specific medications, including clopidogrel, aspirin, celecoxib, loxoprofen, nifedipine, and omeprazole, and both the occurrence of bleeding and abnormalities in coagulation tests ($p < 0.05$). Additionally, interactions of EGb with ticagrelor, etoricoxib, insulin, omeprazole, and domperidone were found to be associated with abnormalities in coagulation tests without affecting physician-reported bleeding.

Table 3: Association of EGb drug interactions with bleeding and abnormal coagulation tests (n=747)

	**The occurrence of bleeding**		**Abnormal coagulant test**	
Variables	OR (CI 5;95%)	P-value	OR (CI 5;95%)	P-value
Weight	1 (0.99;1)	0.607	1 (0.99;1)	0.12
Sex (Ref: Male)	1 (0.97;1.03)	0.733	1.07 (0.99;1.15)	0.071
Age (years)	1 (0.99;1)	0.325	10024 (1;1004)	0.029
BMI (mean±SD)	1 (0.99;1)	0.285	1 (0.99;1.02)	0.198
Occurrence of bleeding (Ref: yes)			1.75 (1.48;2.08)	<0.001
APTT (s)	1 (0.99;1)	0.084	1.03 (1.03;1.04)	<0.001
PT (%)	1.0009 (1.0004;1.001)	0.0005	1005 (1004;1007)	<0.001
Fibrinogen (g/l)	1.04 (1.02;1.05)	<0.001	1.22 (1190;1.25)	<0.001
Abnormal coagulant test (Ref: yes)	1.09 (1.06;1.12)	<0.001		
DDI (Ref: Yes)	1.08 (1.04;1.11)	<0.001	1.49 (1.38;1.58)	<0.001
Number of DDIs	1.06 (1.04;1.09)	<0.001	1.36 (1.3;1.42)	<0.001
Severity of DDIs (Ref: Moderate)	1.01 (0.94;1.08)	0.767	0.813 (0.68;0.96)	0.019

Clopidogrel	1.1 (1.05;1.15)	<0.001	1.58 (1.4;1.77)	<0.001
Aspirin	1.12 (1.07;1.18)	<0.001	1.48 (132;1.66)	<0.001
Ticagrelor	0.96 (0.73;1.27)	0.769	1.83 (0.92;3.61)	0.081
DOAC (enoxaban/rivaroxaban)	1.01 (0.92;1.11)	0.763	1.11 (0.88;1.39)	0.363
Acenocoumarol	0.96 (0.86;1.07)	0.45	1.07 (0.81;1.39)	0.628
Etoricoxib	1 (0.92;1.1)	0.847	1.37 (1.1;1.69)	0.0048
Celecoxib	1.11 (0.99;1.23)	0.055	0.48 (1.15;0.92)	0.0026
Meloxicam	1.06 (0.94;1.2)	0.351	1.23 (0.91;1.67)	0.185
Loxoprofen	1.58 (1.2;2.08)	0.00111	1.83 (0.93;3.61)	0.0807
Nifedipine	1.09 (1;1.18)	0.0371	1.16 (0.95;1.42)	0.139
Insulin	1 (0.92;1.09)	0.925	1.34 (1.09;1.65)	0.0054
Omeprazole	1.10 (1.04;1.15)	0.0003	1.7 (1.51;1.92)	<0.001
Domperidone	1.01 (0.92;1.12)	0.717	1.29 (1.02;1.63)	0.0325

OR: Odds Ratio; CI: Confidence Interval; BMI: Body Mass Index; SD: Standard Deviation; APTT: Activated Partial Thromboplastin Time; PT: Prothrombin Time; DDIs: Drug-Drug Interactions

Discussion

Our findings provide critical insights into the prevalence, severity, and clinical implications of drug interactions involving EGb in conjunction with prescription medications. We observed a considerable frequency of drug interactions, with 12.94% of the prescriptions exhibiting interactions with EGb, which underscores the relevance of monitoring these interactions in clinical practice. A study evaluating dietary supplement use among 458 veteran outpatients receiving prescription medications found that 197 patients (43%) were using at least one dietary supplement concurrently. Of these, 89 patients (45%) had the potential for drug-dietary

supplement interactions. Importantly, most of these interactions (84 or 94%) were classified as non-serious, leading to a prevalence of 6% for potentially severe interactions among those affected, and 3% (5 out of 197) among all patients taking dietary supplements alongside their medications (18).

Among the 195 consecutive patients surveyed in this study, EGb was the predominant herbal medicine. Within the identified herb-drug interactions, eight cases involved interactions between EGb and aspirin (acetylsalicylic acid), whereas one case involved interactions between EGb and trazodone (19). These interactions were predominantly observed with drugs classified as having antiplatelet properties, anticoagulants, and NSAIDs, highlighting a critical area for vigilance among healthcare providers.

Interactions with antiplatelet drugs exhibited the highest prevalence rate and average severity level, with 69 prescriptions for clopidogrel and aspirin documented, reflecting a rate of 2.61%.

In line with our study, the prevalence of concurrent use of EGb with antiplatelet drugs among older adults varies between 5.3% and 88.3% (20). While some case reports indicate potential bleeding risks when combining *G. biloba* with anticoagulants or antiplatelet drugs, controlled clinical studies have not consistently shown significant adverse effects on hemostasis or an increased risk of bleeding (21, 22). Nonetheless, *G. biloba* may enhance the effect of cilostazol on prolonging bleeding time (23), and combining ticlopidine with *G. biloba* extract has been observed to produce greater antiplatelet effects than clopidogrel alone (24). Despite these observations, overall evidence for clinically significant interactions between *G. biloba* and antiplatelet drugs remains limited (25), highlighting the need for healthcare professionals to be

aware of potential interactions and closely monitor patients taking *G. biloba* with antiplatelet medications, especially those at risk of bleeding (26).

Chan et al. identified an association between the risk of hemorrhage and specific patient demographics related to concurrent use of EGb and antiplatelet or anticoagulant agents, particularly patients aged ≥65 and male patients, who were prescribed EGb alone (adjusted odds ratio: 3.8 and 1.4; 95% confidence interval, 2.8–5.2 and 1.1–1.9) (27). Anticoagulant and antiplatelet therapies have been linked to a higher risk of chronic subdural hematoma in the elderly (28, 29). Nonetheless, our investigation did not reveal a correlation between age and risk of bleeding. This lack of an association may stem from the fact that the average age of the patients in our study was 60 years, revealing no discernible differences across age groups. The primary interaction observed was between *G. biloba* and aspirin, which has several significant implications. First, both substances are commonly used by older adults (30). Second, they are readily available as over-the-counter medications, allowing patients to acquire them without the oversight of healthcare professionals. Older individuals often seek *G. biloba* to enhance memory, whereas aspirin is frequently prescribed to manage cardiovascular conditions. There is documented evidence indicating that concurrent use of these substances may increase the risk of bleeding associated with aspirin. The adverse outcomes stemming from this interaction can be severe. A case of spontaneous bleeding linked to the simultaneous intake of *G. biloba* and aspirin has been reported (14). A cross-sectional survey evaluated the use of herbal medicines that may interfere with the effects of antiplatelet or anticoagulant therapy among 250 patients in medical wards. Overall, almost 31% (n=23, N=76) of patients were taking one or more of the specified herbal medicines (ginseng [Panax ginseng], garlic [Allium sativum], and *G. biloba*) thought to interact with antiplatelet or anticoagulant therapy. The study showed that 21% (n=16, N=76) of

patients co-ingested specific herbs with antiplatelet or anticoagulant therapy, of which half were at risk of potential drug–herb interactions (31).

We did not observe a correlation between the interactions involving *G. biloba* and acenocoumarol. While some studies suggest minimal risk, others indicate potential concerns. In vitro studies showed inhibition of CYP2C9 by *G. biloba* extract, but in vivo studies found no significant interactions with CYP2C9 substrates (32). controlled studies using the standardized extract EGb 761 showed no significant impact on hemostasis or adverse effects when combined with aspirin or warfarin (21). Stoddard and colleagues employed text-processing techniques to extract data on *G. biloba* usage and integrated it with prescription records for warfarin from a large clinical database. Their statistical analysis indicated that the concurrent use of *G. biloba* with warfarin significantly increased the risk of adverse bleeding events in patients (hazard ratio = 1.38, 95% CI: 1.20 to 1.58, $p < 0.001$). Disregarding time-at-risk, a bleeding event occurred in 210 (3.1%) patients in the warfarin plus *G. biloba* group compared to 38,408 (5.4%) patients in the warfarin-only group (15).

Interestingly, interactions with medications such as omeprazole, while exhibiting a relatively high frequency, were classified as mild. Eighteen healthy Chinese participants were recruited to explore the potential herb-drug interactions between EGb and omeprazole, a commonly prescribed CYP2C19 substrate, across different CYP2C19 genotypes. The findings from this study indicate that EGb can induce omeprazole hydroxylation, with the extent of induction varying based on the individual's CYP2C19 genotype. Additionally, the concurrent administration of EGb decreased the renal clearance of 5-hydroxyomeprazole. These results suggest that coadministration of EGb with omeprazole or other CYP2C19 substrates may significantly affect their efficacy (33).

Nonetheless, these interactions still warrant attention owing to their potential impact on coagulation profiles. Further analysis revealed a significant association between drug interactions involving *G. biloba* and the occurrence of bleeding and coagulation test abnormalities. Clinical studies have shown that *G. biloba* does not affect the APTT, PT, or fibrinogen indices (34). However, 15 case reports published in the literature have delineated the temporal correlation between *G. biloba* administration and the occurrence of bleeding events (12). Most of these cases involved severe medical conditions, with eight incidents specifically involving intracranial bleeding. Nevertheless, other risk factors for bleeding were identified among the 13 case reports. Six reports documented the cessation of *G. biloba* intake, which resulted in the absence of recurrent bleeding episodes. Additionally, three reports indicated elevated bleeding times in patients with *G. biloba* intake (12).

Our findings underscore the need for clinicians to be vigilant in assessing potential interactions, particularly when prescribing *G. biloba* in conjunction with medications known to increase bleeding risk. Additionally, the study revealed no correlation between the severity of drug interactions involving EGb and the incidence of bleeding, as indicated by an odds ratio (OR) of 1.01 (95% CI: 0.94; 1.08) and a p-value of 0.767, demonstrating no significant association. Conversely, a significant effect was noted regarding abnormalities in coagulation test results, with an OR of 0.813 (95% CI: 0.68; 0.96) and a p-value of 0.019. Research on the interactions between *G. biloba* and anticoagulant and antiplatelet drugs presents mixed findings. Potential mechanisms of interaction may include pharmacodynamic effects or the induction of hepatic cytochrome P450 enzymes (35). The severity of these interactions varies from moderate to severe (26). While some cases report an increased risk of bleeding, pharmacokinetic studies show inconsistent results (36). This emphasizes the importance of considering the severity of the

interactions between clinical decision-making and patient management. The observed associations between specific medications and the occurrence of bleeding and coagulation abnormalities further highlight the need for careful evaluation and monitoring of patients receiving *G. biloba* therapy. Clopidogrel, aspirin, celecoxib, loxoprofen, nifedipine, and omeprazole were among the medications significantly associated with these outcomes, warranting particular attention in clinical practice.

Our study had some limitations. First, the study sample was derived from patients who were prescribed *G. biloba*; this may have introduced selection bias. Patients who choose to use or are prescribed *G. biloba* may possess different characteristics compared to those who do not use the supplement, potentially impacting the generalizability of our findings. Second, despite our efforts to collect comprehensive medical records, some data, particularly laboratory tests, including APTT, PT, and fibrinogen levels, may have been missing or incomplete. This incompleteness could compromise the accuracy and reliability of our results, leading to a potential underestimation or overestimation of the prevalence of drug interactions and bleeding risk. Additionally, our study may not account for all potential confounding variables that could influence the bleeding risk and abnormal coagulation profiles. Factors such as comorbidities, concurrent medications, lifestyle habits, and dietary patterns, which may have influenced the associations observed in our study, were not thoroughly assessed. These limitations should be considered when interpreting the results of this study.

Conclusion

In conclusion, this study provides valuable insights into the prevalence and implications of drug interactions involving EGb on bleeding risk and coagulation profiles. A significant prevalence of drug interactions was observed, primarily with antiplatelets, anticoagulants, and NSAIDs. Notably, interactions with anticoagulants such as direct oral anticoagulants (DOACs) and acenocoumarol were not significant in this study. Among these interactions, antiplatelet medications like clopidogrel and aspirin exhibited the highest prevalence, while omeprazole was frequently involved, though with mild severity. The findings revealed a strong association between clinical bleeding and abnormal coagulation, highlighting notable correlations between EGb drug interactions, bleeding risk, and coagulation abnormalities. Interestingly, while the severity of EGb drug interactions did not correlate with bleeding risk, it did impact coagulation test results. Specific medications, including clopidogrel, aspirin, celecoxib, loxoprofen, nifedipine, and omeprazole, were significantly linked to both bleeding risk and abnormal coagulation. Overall, our findings underscore the importance of considering EGb drug interactions in bleeding risk assessments and coagulation management, highlighting the need for further research in this area to optimize patient safety and outcomes.

Acknowledgments

We would like to express our gratitude to all the physicians and nurses in the Outpatient Clinic and Laboratory Department of Hai Phong International Hospital for their assistance in patient recruitment and clinical laboratory testing for our study. Furthermore, we greatly appreciate the

invaluable support provided by Hai Phong University of Medicine and Pharmacy for our Biomedical - Pharmaceutical Sciences Research Group in this endeavor.

References

1. McKenna DJ, Jones K, Hughes K. Efficacy, safety, and use of ginkgo biloba in clinical and preclinical applications. Altern Ther Health Med. 2001;7(5):70-86, 8-90.

2. Singh SK, Srivastav S, Castellani RJ, Plascencia-Villa G, Perry G. Neuroprotective and Antioxidant Effect of Ginkgo biloba Extract Against AD and Other Neurological Disorders. Neurotherapeutics. 2019;16(3):666-74.

3. Ginkgo. Drugs and Lactation Database (LactMed(R)). Bethesda (MD)2006.

4. Tchantchou F, Xu Y, Wu Y, Christen Y, Luo Y. EGb 761 enhances adult hippocampal neurogenesis and phosphorylation of CREB in transgenic mouse model of Alzheimer's disease. FASEB J. 2007;21(10):2400-8.

5. Yoshitake T, Yoshitake S, Kehr J. The Ginkgo biloba extract EGb 761(R) and its main constituent flavonoids and ginkgolides increase extracellular dopamine levels in the rat prefrontal cortex. Br J Pharmacol. 2010;159(3):659-68.

6. Marcilhac A, Dakine N, Bourhim N, Guillaume V, Grino M, Drieu K, et al. Effect of chronic administration of Ginkgo biloba extract or Ginkgolide on the hypothalamic-pituitary-adrenal axis in the rat. Life Sci. 1998;62(25):2329-40.

7. Le Bars PL, Katz MM, Berman N, Itil TM, Freedman AM, Schatzberg AF. A placebo-controlled, double-blind, randomized trial of an extract of Ginkgo biloba for dementia. North American EGb Study Group. JAMA. 1997;278(16):1327-32.

8. Birks J, Grimley Evans J. Ginkgo biloba for cognitive impairment and dementia. Cochrane Database Syst Rev. 2009(1):CD003120.

9. Tan MS, Yu JT, Tan CC, Wang HF, Meng XF, Wang C, et al. Efficacy and adverse effects of ginkgo biloba for cognitive impairment and dementia: a systematic review and meta-analysis. J Alzheimers Dis. 2015;43(2):589-603.

10. Unger M. Pharmacokinetic drug interactions involving Ginkgo biloba. Drug Metab Rev. 2013;45(3):353-85.

11. Kellermann AJ, Kloft C. Is there a risk of bleeding associated with standardized Ginkgo biloba extract therapy? A systematic review and meta-analysis. Pharmacotherapy. 2011;31(5):490-502.

12. Bent S, Goldberg H, Padula A, Avins AL. Spontaneous bleeding associated with ginkgo biloba: a case report and systematic review of the literature: a case report and systematic review of the literature. J Gen Intern Med. 2005;20(7):657-61.

13. Ang-Lee MK, Moss J, Yuan CS. Herbal medicines and perioperative care. JAMA. 2001;286(2):208-16.

14. Rosenblatt M, Mindel J. Spontaneous hyphema associated with ingestion of Ginkgo biloba extract. N Engl J Med. 1997;336(15):1108.

15. Stoddard GJ, Archer M, Shane-McWhorter L, Bray BE, Redd DF, Proulx J, et al. Ginkgo and Warfarin Interaction in a Large Veterans Administration Population. AMIA Annu Symp Proc. 2015;2015:1174-83.

16. Kohler S, Funk P, Kieser M. Influence of a 7-day treatment with Ginkgo biloba special extract EGb 761 on bleeding time and coagulation: a randomized, placebo-controlled, double-blind study in healthy volunteers. Blood Coagul Fibrinolysis. 2004;15(4):303-9.

17. R: TR. A language and environment for statistical computing. R Foundation for Statistical Computing. 2005.

18. Peng CC, Glassman PA, Trilli LE, Hayes-Hunter J, Good CB. Incidence and severity of potential drug-dietary supplement interactions in primary care patients: an exploratory study of 2 outpatient practices. Arch Intern Med. 2004;164(6):630-6.

19. Dergal JM, Gold JL, Laxer DA, Lee MS, Binns MA, Lanctot KL, et al. Potential interactions between herbal medicines and conventional drug therapies used by older adults attending a memory clinic. Drugs Aging. 2002;19(11):879-86.

20. Agbabiaka TB, Wider B, Watson LK, Goodman C. Concurrent Use of Prescription Drugs and Herbal Medicinal Products in Older Adults: A Systematic Review. Drugs Aging. 2017;34(12):891-905.

21. Bone KM. Potential interaction of Ginkgo biloba leaf with antiplatelet or anticoagulant drugs: what is the evidence? Mol Nutr Food Res. 2008;52(7):764-71.

22. Gaby AR, editor Ginkgo Biloba Extract: A Review1996.

23. Aruna D, Naidu MU. Pharmacodynamic interaction studies of Ginkgo biloba with cilostazol and clopidogrel in healthy human subjects. Br J Clin Pharmacol. 2007;63(3):333-8.

24. Hong JM, Shin DH, Lim YA, Lee JS, Joo IS. Ticlopidine with Ginkgo Biloba extract: a feasible combination for patients with acute cerebral ischemia. Thromb Res. 2013;131(4):e147-53.

25. Abad MJ, Bedoya LM, Bermejo P. An update on drug interactions with the herbal medicine Ginkgo biloba. Curr Drug Metab. 2010;11(2):171-81.

26. Tsai HH, Lin HW, Lu YH, Chen YL, Mahady GB. A review of potential harmful interactions between anticoagulant/antiplatelet agents and Chinese herbal medicines. PLoS One. 2013;8(5):e64255.

27. Chan AL, Leung HW, Wu JW, Chien TW. Risk of hemorrhage associated with co-prescriptions for Ginkgo biloba and antiplatelet or anticoagulant drugs. J Altern Complement Med. 2011;17(6):513-7.

28. De Bonis P, Trevisi G, de Waure C, Sferrazza A, Volpe M, Pompucci A, et al. Antiplatelet/anticoagulant agents and chronic subdural hematoma in the elderly. PLoS One. 2013;8(7):e68732.

29. Nathan S, Goodarzi Z, Jette N, Gallagher C, Holroyd-Leduc J. Anticoagulant and antiplatelet use in seniors with chronic subdural hematoma: Systematic review. Neurology. 2017;88(20):1889-93.

30. Gold JL, Laxer DA, Dergal JM, Lanctot KL, Rochon PA. Herbal-drug therapy interactions: a focus on dementia. Curr Opin Clin Nutr Metab Care. 2001;4(1):29-34.

31. Saw JT, Bahari MB, Ang HH, Lim YH. Potential drug-herb interaction with antiplatelet/anticoagulant drugs. Complement Ther Clin Pract. 2006;12(4):236-41.

32. Mohutsky MA, Anderson GD, Miller JW, Elmer GW. Ginkgo biloba: evaluation of CYP2C9 drug interactions in vitro and in vivo. Am J Ther. 2006;13(1):24-31.

33. Yin OQ, Tomlinson B, Waye MM, Chow AH, Chow MS. Pharmacogenetics and herb-drug interactions: experience with Ginkgo biloba and omeprazole. Pharmacogenetics. 2004;14(12):841-50.

34. Bal Dit Sollier C, Caplain H, Drouet L. No alteration in platelet function or coagulation induced by EGb761 in a controlled study. Clin Lab Haematol. 2003;25(4):251-3.

35. Taki Y, Yokotani K, Yamada S, Shinozuka K, Kubota Y, Watanabe Y, et al. Ginkgo biloba extract attenuates warfarin-mediated anticoagulation through induction of hepatic cytochrome P450 enzymes by bilobalide in mice. Phytomedicine. 2012;19(2):177-82.

36. Li J, Liang Q, Sun G. Interaction between Traditional Chinese Medicine and Anticoagulant/Antiplatelet Drugs. Curr Drug Metab. 2019;20(9):701-13.

Click here to access/download;Figure;Figure 1.tif

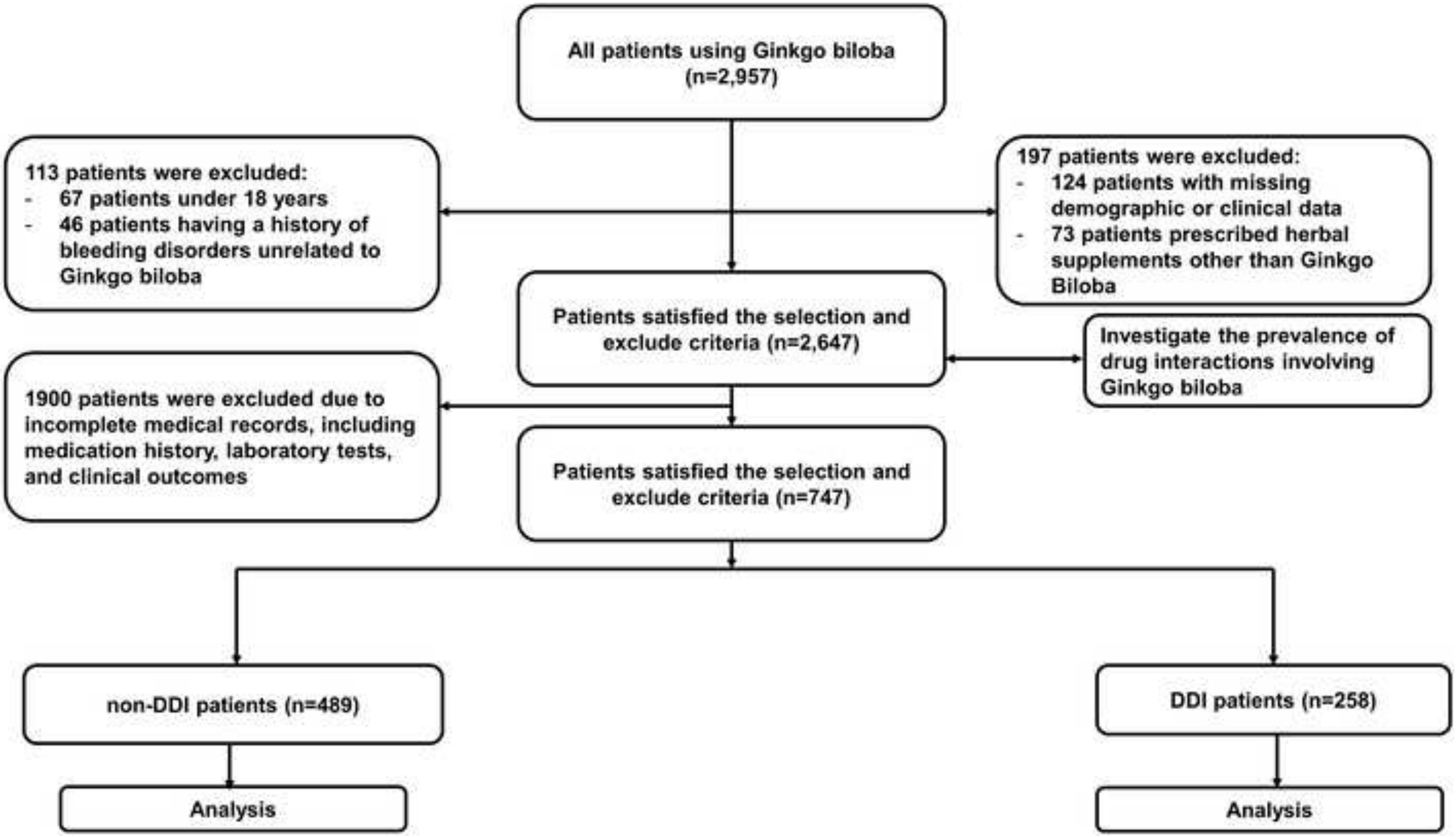

Study data

Click here to access/download
Supporting Information
data_ddi.xlsx

original ethics approval letter

Click here to access/download

Supporting Information

IRB_origional_file.pdf

Click here to access/download
Supporting Information
IRB_translated.pdf

Style Definition: Normal: Line spacing: Double

Impact of Ginkgo biloba drug interactions on bleeding risk and coagulation profiles: A comprehensive analysis

Short title: Impact of Ginkgo Biloba Interactions on Bleeding Risk and Coagulation

Ngo Thi Quynh Mai[1], Nguyen Viet Hieu[1], Tran Thi Ngan[1,2], Tran Van Anh[1,2], Pham Van Linh[3], Nguyen Thi Thu Phuong[1,2]

[1] Faculty of Pharmacy, Hai Phong University of Medicine and Pharmacy, Hai Phong 180000, Vietnam

[2] Department of Pharmacy, Hai Phong International Hospital, Hai Phong 180000, Vietnam

[3] Department of Pathology and Immunology, Hai Phong University of Medicine and Pharmacy, Hai Phong 180000, Vietnam

* Corresponding author:

Email: nttphuong@hpmu.edu.vn (NTTP)

Abstract

This retrospective observational study was conducted to investigate the prevalence and implications of drug interactions involving *Ginkgo biloba* extract on bleeding risk and coagulation profiles. Our analysis utilized data from patients admitted to Hai Phong International Hospital between January 2022 and December 2023. Inclusion criteria consisted of patients aged 18 years and above, those prescribed *Ginkgo biloba* extract alone or in combination with other medications, and the availability of complete medical records, including medication history, laboratory tests, and clinical outcomes. Among the 2,647 prescriptions that met the inclusion criteria, 342 exhibited drug interactions with a prevalence rate of 12.94%. ~~Notably,~~ *Ginkgo biloba* extract primarily interacted~~s~~ with antiplatelets, anticoagulants, and nonsteroidal anti-inflammatory drugs, with Clopidogrel and Aspirin exhibiting the highest prevalence rates of 2.61% each. However, the interactions with anticoagulants, particularly direct oral anticoagulants and acenocoumarol, were not significant~~ in our analysis~~. Omeprazole was a frequently interacting drug (2.34%) of mild severity. Among the 747 patients analyzed for bleeding disorders, 31 (4.15%) exhibited bleeding symptoms. Correlation analysis indicated a strong association between clinical bleeding and abnormal coagulation results (OR, 1.75; $p < 0.001$). Moreover, significant correlations were found between *Ginkgo biloba* extract drug interactions and the bleeding risk (OR: 1.08, $p < 0.001$) and abnormal coagulation (OR: 1.49, $p < 0.001$). The severity of *Ginkgo biloba* extract drug interactions did not correlate with bleeding risk (OR: 1.01, $p = 0.767$) but influenced abnormal coagulation test results (OR: 0.813, $p = 0.019$). Specific medications, including clopidogrel, aspirin, celecoxib, loxoprofen, nifedipine, and omeprazole, were significantly associated with the risk of bleeding and abnormal coagulation ($p < 0.05$).

Formatted: Font: Italic

Interactions with ticagrelor, etoricoxib, insulin, omeprazole, and domperidone were associated with abnormal coagulation tests without affecting the reported bleeding. These findings emphasize the importance of considering *Ginkgo biloba* extract drug interactions in bleeding risk assessment and coagulation management.

Keywords: Ginkgo biloba; drug interactions; bleeding risk; anticoagulants; coagulation profiles

Introduction

Ginkgo biloba extract, a herbal supplement derived from the leaves of the species *Ginkgo biloba (G. biloba)*, has gained popularity worldwide owing to its perceived health benefits, including cognitive enhancement and peripheral circulation improvement (1, 2). *G. biloba* leaves contain two primary active ingredients: terpene lactones (including ginkgolides and diterpenes) and Ginkgo flavone glycosides (containing ginkgetin, bilobetin, and sciadopitysin), which are present in varying concentrations (3). Known for its positive effects on mental focus, *G. biloba* leaf extract (EGb) is recognized as one of the most widely used health supplements. The standardized version, EGb 761, is frequently employed in research exploring its effects. Studies have shown that EGb affects various neurotransmitter systems and brain structures, particularly evident in animal research*G. biloba* leaf extract (EGb) is widely recognized as one of the most popular health supplements globally due to its benefits for mental focus. EGb 761, the standardized EGb, is commonly used in studies investigating its effects. Research has indicated that EGb influences several neurotransmitter pathways and brain structures, particularly those observed in animal studies (4, 5). EGb 761 reduces stress-induced corticosterone hypersecretion in rats by decreasing the number of peripheral adrenal benzodiazepine receptors (6). However, the efficacy of EGb 761 in treating dementia has been a topic of debate, with conflicting findings from

Formatted: Font: 12 pt
Formatted: Font: 12 pt, Bold
Formatted: Font: (Default) Times New Roman
Formatted: Font: (Default) Times New Roman
Formatted: Font: (Default) Times New Roman
Formatted: Font: (Default) Times New Roman
Formatted: Font: (Default) Times New Roman
Formatted: Font: (Default) Times New Roman
Formatted: Font: (Default) Times New Roman
Formatted: Font: (Default) Times New Roman
Formatted: Font: 12 pt, Bold, Vietnamese
Formatted: Font: (Default) Times New Roman
Formatted: Font: (Default) Times New Roman, Italic
Formatted: Font: (Default) Times New Roman

various studies. A 52-week randomized, double-blind, placebo-controlled trial involving 309 patients suggested that EGb 761 was safe and, albeit modestly, appeared to stabilize and improve cognitive performance and social functioning in patients with dementia for 6 months to 1 year (7). Systematic reviews conducted in 2009 and 2018, comprising 36 and 38 trials, respectively, highlighted the safety of EGb but did not find sufficient evidence to support its clinical benefit in patients with cognitive impairment and dementia (8). Conversely, a 2015 systematic review of nine trials suggested that EGb 761 at a dose of 240 mg/day could slow the decline in cognition, function, behavior, and overall global change at 22–26 weeks, particularly in patients with neuropsychiatric symptoms (9). Overall, EGb is regarded as safe and generally well-tolerated, with a maximum recommended dose of 240 mg/day (10). Mild side effects may include headache, heart palpitations, gastrointestinal issues, constipation, and allergic skin reactions (3). Although a systematic review and meta-analysis found no significant effect of EGb on prothrombin time, activated partial thromboplastin time, or platelet aggregation, several case reports have noted a temporal link between EGb use and bleeding events, including severe intracranial bleeding (11, 12).

Although is commonly used, it carries certain risks, particularly regarding its potential interactions with prescription medications. Such interactions may result in complications like bleeding disorders and abnormal coagulation profiles ~~Despite its widespread use, EGb has risks, particularly concerning its potential to interact with prescription medications, leading to adverse effects such as bleeding disorders and abnormal coagulation profiles~~ (13). Therefore, it is important to exercise caution when administering EGb to patients with bleeding disorders or those using NSAIDs, antiplatelet drugs, or anticoagulants ~~Therefore, caution should be exercised when using EGb in patients with bleeding disorders or those taking NSAIDs, antiplatelets, or~~

anticoagulants. One case report described an instance of spontaneous bleeding from the iris into the anterior chamber of the eye in an elderly patient who was concurrently using aspirin and Egb (14). Additionally, a 2015 study conducted in a large Veterans Administration population found a correlation between the coadministration of warfarin and EGb and an increased risk of bleeding events (15).

Recently, several case reports have indicated a possible association between hemorrhagic complications and the use of EGb preparations. In light of these concerns, a trial was conducted to assess the effects of the *G. biloba* special extract, EGb 761, on hemostatic parameters. The results revealed that none of the 29 coagulation and bleeding parameters evaluated showed any evidence of EGb 761 inhibiting blood coagulation or platelet aggregationIn recent years, multiple case reports have raised concerns about a potential causal link between hemorrhagic complications and the use of EGb preparations. Consequently, a trial was conducted to examine the impact of the Ginkgo biloba special extract, EGb 761, on hemostatic parameters. This study indicated that, among the 29 coagulation and bleeding parameters assessed, none showed any evidence of inhibition of blood coagulation or platelet aggregation by EGb 761. Furthermore, the study did not reveal any evidence to substantiate the causal relationship between EGb administration of EGb 761 and hemorrhagic complications (16). However, the concomitant use of EGb with prescription drugs has raised concerns among healthcare professionals owing to the potential for pharmacokinetic and pharmacodynamic interactions. These interactions can alter the efficacy and safety profiles of EGb and co-administered medications, posing significant challenges in clinical management. Concerning perioperative use, there is insufficient evidence regarding the risks associated with EGb use. However, one study suggested that physicians should consider discontinuing EGb for at least 36 h before a planned surgical procedure (13).

Formatted: Font color: Auto, Pattern: Clear

Formatted: Normal (Web), Left, Line spacing: single

Formatted: Font color: Auto, Pattern: Clear

~~Due to a lack of data on its safety and efficacy, it is contraindicated for pregnant women, nursing mothers, or infants (3). Furthermore, caution is warranted in epileptic patients or those prone to seizures, as Ginkgo toxin, primarily present in Ginkgo seeds but also found in Ginkgo leaves, may lower the seizure threshold (17, 18).~~

~~Given the increasing prevalence of polypharmacy and the widespread use of complementary and alternative medicines, understanding the impact of EGb drug interactions on bleeding risk and coagulation profiles is of paramount importance. However, despite the growing recognition of these interactions, there is a paucity of comprehensive analyses evaluating their prevalence, severity, and clinical implications.~~

Formatted: Indent: Left: 0"

Through a retrospective observational study design, we sought to clarify the frequency and severity of drug interactions involving EGb, specifically in relation to bleeding risk and abnormalities in coagulation profiles in a real-world clinical context. The results of this study could inform clinical decision-making, improve patient care, and enhance medication safety for individuals undergoing EGb therapy in combination with prescription medications~~Through a retrospective observational study design, we aimed to elucidate the frequency and severity of EGb drug interactions, with a particular focus on their association with bleeding risk and abnormalities in coagulation profiles in a real-world clinical setting. Ultimately, the findings of this study have the potential to guide clinical decision-making, optimize patient care, and enhance medication safety in individuals receiving EGb therapy concurrently with prescription medications~~.

Formatted: Normal (Web), Left, Line spacing: single

Formatted: Font: 11 pt, Font color: Black

Materials and methods

Study design

This retrospective observational study analyzed data collected from the medical records of patients admitted to Hai Phong International Hospital (Hai Phong, Vietnam) between January 2022 and December 2023. Data were accessed for study purposes on February 15, 2024.

Inclusion criteria:

– Patients aged 18 years and older

– Patients prescribed EGb either alone or in combination other prescription medications

– Availability of complete medical records, including medication history, laboratory tests, and clinical outcomes.

This criterion was specifically applied to assess the impact of EGb interactions on bleeding occurrences or abnormal coagulation tests.

~~– Patients aged 18 years and above~~

~~– Patients prescribed EGb alone or in combination with other prescription drugs~~

~~– Availability of complete medical records, including medication history, laboratory tests, and clinical outcomes. This criterion was only applied to investigate the effect of EGb interactions on the occurrence of bleeding or abnormal coagulation tests.~~

Exclusion criteria:

– Patients with incomplete medical records

– Patients with a history of bleeding disorders unrelated to medication use

– Patients prescribed herbal supplements other than EGb

- Patients with missing demographic or clinical data

Data extraction:

A structured data extraction form was used to collect the following information:

- Patient demographics (age, gender, weight, body mass index [BMI])
- Medication history, including EGb and other prescription drugs
- Laboratory tests, including activated partial thromboplastin time (APTT), prothrombin time (PT), and fibrinogen levels
- Clinical outcomes such as bleeding events and diagnosis of bleeding disorders
- Severity classification of drug interactions based on established criteria

Identification of drug interactions:

Drug interactions involving EGb were identified using reputable drug interaction databases such as UpToDate/Micromedex/Drugs.com. Interactions were classified based on severity (mild, moderate, or severe) and documented adverse effects, particularly bleeding disorders and abnormal coagulation profiles.

Statistical analyses

Descriptive statistics were used to summarize the patient demographics, medication profiles, and clinical outcomes. The prevalence of drug interactions with EGb was calculated as the proportion of patients with documented interactions. Chi-square tests ~~or Fisher's exact tests~~ were used to assess associations between categorical variables, whereas t-tests ~~or Mann–Whitney U~~ tests were used for continuous variables. Logistic regression analysis was performed to identify the factors

associated with bleeding events and abnormal coagulation test results. Statistical significance was determined~~set~~ at $p < 0.05$. The logistic regression model was built using the R statistical software, version 3.2.4 (A Language and Environment for Statistical Computing, Vienna, Austria) (17).

Ethical considerations

This study adhered to the ethical principles outlined in the Declaration of Helsinki. Patient confidentiality and privacy were maintained throughout the data collection and analysis. Institutional Review Board (IRB.23.128) approval was obtained before the commencement of data collection and analysis. All patients provided verbal consent for the use of their data in research at the time of their initial treatment. This consent included an explanation of the potential use of their anonymized data in future research studies.

Results

To calculate the frequency and rate of drug interactions between EGb and other prescription drugs, we selected 2,647 prescriptions that met the inclusion and exclusion criteria. Among these, 342 prescriptions exhibited drug interactions, resulting in a prevalence of 12.94%. The study diagram is presented in Fig 1.

Figure 1. Study diagram

The interaction between EGb and conventional drugs is presented in Table 1. EGb interacts predominantly with antiplatelet drugs, anticoagulants, and NSAIDs. Interactions with antiplatelet drugs manifested the highest prevalence rate, coupled with an average severity level.

Specifically, interactions with clopidogrel and aspirin were documented in 69 prescriptions, representing a rate of 2.61%.

Furthermore, interactions between EGb and anticoagulants have also garnered significant attention owing to their notable prevalence rates. Noteworthy interactions were observed with direct oral anticoagulants (DOACs), documented in 20 prescriptions (18%), and with acenocoumarol, noted in 13 prescriptions (0.49%). According to UpToDate/Micromedex, interactions between EGb and agents with antiplatelet properties/anticoagulants may potentiate adverse or toxic effects, including bleeding.

Within the spectrum of interactions between EGb and other drugs, omeprazole emerged at a notable frequency, as documented in 62 prescriptions, constituting 2.34% of the observed interactions. Despite the relatively high rate, the severity of this interaction pair was considered mild.

Out of 2,647 patients prescribed EGb preparations, 747 were eligible for Part 2 of the study, focusing on the relationship between EGb drug interactions and bleeding disorders. Among these, 489 patients without drug interactions formed the control group, while 258 patients had drug interactions. The characteristics of these patients, including weight, age, and sex, are detailed in Table 2. Notably, 31 patients (4.15%) were diagnosed with bleeding disorders or symptoms. The clinical characteristics and drug interactions of this subset were analyzed, revealing a mean activated partial thromboplastin time (APTT) of 57.25 ± 9.34 seconds, a prothrombin time (PT) of 110.71 ± 26.18%, and a fibrinogen level of 3.56 ± 1.25 g/L~~Of the 2,647 patients prescribed EGb preparation, 747 met the criteria for participation in Part 2 of the study aimed at exploring the relationship between drug interactions involving EGb and bleeding~~

disorders. Of the 747 patients treated with EGb preparation, 489 individuals without drug interactions were included in the control group, whereas the group with drug interactions comprised 258 patients. Table 2 presents the characteristics of these patients, including weight, age, and sex, categorized into two groups: those with drug interactions involving EGb and other drugs and those without such interactions. Among them, 31 (4.15%) were diagnosed with bleeding disorders or symptoms. The clinical characteristics and drug interactions between EGb and other medications in this subset of patients were meticulously analyzed. The mean (±SD) of APTT was found to be 57.25 ± 9.34 seconds, PT (%) was 110.71 ± 26.18, and fibrinogen level was 3.56 ± 1.25 g/L.

To clarify the relationship between EGb drug interactions and bleeding disorders, a control group of 484 patients without drug interactions was established, alongside 258 patients who had interactions with other medications. Among these patients, the interaction pairs were distributed as follows: 185 patients had one interaction (61.24%), 64 had two (24.81%), seven had three (2.71%), and two had four (0.77%). Of the 258 patients with drug interactions, 226 (87.6%) experienced moderate severity interactions, while 32 (12.4%) had mild severity interactions, as outlined in Table 2.

To further elucidate the relationship between drug interactions involving EGb and bleeding disorders, a control group of 484 patients without drug interactions was selected. In addition, 258 patients exhibited an interaction between EGb and other drugs. The distribution of interaction pairs was as follows: 185 patients had one interaction pair (61.24%), 64 patients had two interaction pairs (24.81%), seven patients had three interaction pairs (2.71%), and two patients had four interaction pairs (0.77%). Of the 258 patients experiencing drug-drug interactions

involving EGb, 226 individuals were identified with moderate severity interactions (87.6%), while only 32 patients exhibited mild severity interactions (12.4%) (Table 2).

Table 1: Interaction between EGb and conventional drugs (n=2,647)

Drug class	Prescribed drug	Number of DDIs	Frequency (%)	Severity	Mechanism	Consequence	Reference
Agents with antiplatelet properties	Clopidogrel	69	2.61	Moderate	Many herbal products have been shown to inhibit platelet function, prolong bleeding time, or contribute to bleeding events in people. Concomitant use of these herbal products with drugs that have a similar pharmacologic potential may increase the risk of bleeding.	Enhance the adverse/toxic effects of agents with antiplatelet properties. Bleeding may occur	UpToDate
	Aspirin	69	2.61	Moderate			
	Ticagrelor	2	0.08	Moderate			
Anticoagulants	DOAC (enoxaban/ rivaroxaban)	18	0.68	Moderate	Many herbal products possess the ability to cause or potentiate bleeding (inhibit clotting/coagulation or primary hemostasis) by one of several mechanisms. They may inhibit platelet aggregation, inhibit cyclooxygenase activity, interfere with one or more components of the coagulation cascade, or increase bleeding risk by another mechanism. The concomitant use of such herbs with other herbs or drugs possessing a similar pharmacologic potential may increase the risk of bleeding.	Enhance the adverse/toxic effects of anticoagulants. Bleeding may occur.	UpToDate
	Acenocoumarol	13	0.49	Moderate			

Drug class	Prescribed drug	Number of DDIs	Frequency (%)	Severity	Mechanism	Consequence	Reference
Nonsteroidal anti-inflammatory drugs (NSAIDs)	Etoricoxib	20	0.76	Moderate	Many herbal products have been shown to inhibit platelet function, prolong bleeding time, or contribute to bleeding events in people. Concomitant use of these herbal products with drugs that have a similar pharmacologic potential (such as NSAIDs) may increase the risk of bleeding.	Enhance the adverse/toxic effects of NSAIDs. Bleeding may occur	UpToDate
	Celecoxib	14	0.53	Moderate			
	Meloxicam	10	0.38	Moderate			
	Loxoprofen	2	0.08	Moderate			
Other	Nifedipine	24	0.91	Moderate	Inhibition of cytochrome P450 3A4 by *G. biloba*~~Ginkgo~~	Concurrent use of *G. biloba* and nifedipine may result in an increased risk of nifedipine side effects.	UpToDate
	Insulin	22	0.83	Moderate	*G. biloba*~~Ginkgo~~ may increase pancreatic beta-cell function	Concurrent use of *G. biloba* and insulin may result in altered insulin effectiveness.	Micromedex
	Omeprazol	62	2.34	Minor	The mechanism of this potential interaction is	Decrease the serum	Microme

Formatted: Font: 10 pt

Formatted: Font: 10 pt

Drug class	Prescribed drug	Number of DDIs	Frequency (%)	Severity	Mechanism	Consequence	Reference
	e				unknown. While *G. biloba*-mediated induction of CYP2C19 has been proposed as a potential mechanism, the large effect seen in CYP2C19 poor metabolizers combined with a lack of effects seen with voriconazole and diazepam (substrates of CYP2C19) in other studies [3,4] makes the role of CYP2C19 induction in this interaction questionable. Additionally, because natural products have variable constituents, it is unknown if a particular formulation or dose is more susceptible to this interaction.	concentration of omeprazole	dex
	Domperidone	17	0.64	Minor	Inhibition of CYP3A4-mediated domperidone metabolism	Concurrent use of domperidone and *G. biloba* may result in increased domperidone exposure and an	Micromedex

Drug class	Prescribed drug	Number of DDIs	Frequency (%)	Severity	Mechanism	Consequence	Reference
						increased risk of QT prolongation.	
Total		342	12.94				

DDIs: Drug-drug interactions; DOAC: Direct oral anticoagulant; NSAIDs: Nonsteroidal anti-inflammatory drugs.

Table 2: Clinical characteristics and drug interactions of the patient group to evaluate the relationship between DDI of EGb - bleeding/coagulopathy (n=747)

	**No DDI (n=489)**	**DDI (n=258)**	**Total (n=747)**
Weight (kg, mean±SD)	57.14±9.44	57.46±9.15	57.25±9.34
Age (years, mean±SD)	54.26±15.79	63.08±13.93	57.31±15.74
Sex (Ref: female) (n,%)	331 (67.69%)	172 (66.67%)	503 (67.34%)
BMI (mean±SD)	22.62±2.81	23.09±2.78	22.78±2.81
Symptom of bleeding (Ref: yes) n,%)	8(1.64%)	23(8.91%)	31(4.15%)
APTT (s) (mean±SD)	33.3±4.56	34.11±5.45	33.78±5.12
PT (%, mean±SD)	102.2±17.31	126.83±31.92	110.71±26.18
Fibrinogen (g/l, mean±SD)	3.19±0.7	5.24±1.78	3.55±1.25
Abnormal coagulant test (n,%)	127 (25.97%)	169 (65.5%)	296 (39.63%)
Number of DDIs (n,%)			
One interaction pair		185 (71.71%)	185 (24.77%)
Two interaction pairs		64 (24.81%)	64 (8.57%)
Three interaction pairs		7 (2.71%)	7 (0.94%)
Four interaction pairs		2 (0.78%)	2 (0.27%)
Severity of DDI (n=258)			
Moderate		32 (12.4%)	32 (4.28%)
Minor		226 (87.6%)	715 (95.72%)

DDIs: Drug-Drug Interactions; SD: Standard Deviation; BMI: Body Mass Index; APTT: Activated Partial Thromboplastin Time; PT: Prothrombin Time

Investigate how clinical variables and drug interaction characteristics are associated with the incidence of clinically documented bleeding and abnormalities in coagulation test results among

Formatted: Font: (Default) Times New Roman

747 patients. The results indicate that weight and BMI did not significantly impact the occurrence of recorded bleeding or abnormalities in coagulation test results (p > 0.05, as shown in Table 3). We conducted a correlation analysis to examine the relationship between clinical variables and drug interaction characteristics and the occurrence of clinically documented bleeding and abnormalities in coagulation test results among 747 patients. The findings, as presented in Table 3, indicate that weight and BMI did not significantly influence the occurrence of clinically recorded bleeding or abnormalities in coagulation tests (p > 0.05). Although sex did not demonstrate a substantial association with the occurrence of bleeding, it exhibited a slight correlation with abnormalities in coagulation test results, with an odds ratio (OR) of 1.07 (95% confidence interval (6): 0.99; 1.15) (-p = 0.071).

The study found a significant association between clinical bleeding cases and abnormal coagulation results, with an odds ratio (OR) of 1.75 (95% CI: 1.48; 2.08), indicating a strong correlation (p < 0.001). Each coagulation test APTT, PT, and fibrinogen levels demonstrated a strong relationship with the occurrence of clinical bleedingOur analysis revealed a significant association between cases of clinical bleeding and abnormal coagulation results, with an OR of 1.75 (95% CI: 1.48; 2.08), indicating a strong correlation (p < 0.001). Each coagulation test, including APTT, PT, and fibrinogen level, demonstrated a close relationship with the manifestation of clinical bleeding.

Formatted: Font: (Default) Times New Roman

Formatted: Justified, Line spacing: single

Formatted: Font: (Default) Times New Roman

Formatted: Font: (Default) Times New Roman

Furthermore, theour study revealed a noteworthy link between drug interactions involving EGb and prescription medications and the occurrence of bleeding and abnormal coagulation test results. The odds ratios for these associations were found to be 1.08 (95% CI: 1.04; 1.11) and 1.49 (95% CI: 1.38; 1.58), respectively, implying a significant correlation (p < 0.001). These

findings underscore the clinical relevance of drug interactions, particularly those involving EGb, in the context of bleeding risk and abnormal coagulation profiles.

The study found no correlation between the severity of drug interactions involving EGb and the occurrence of bleeding, with an odds ratio (OR) of 1.01 (95% CI: 0.94; 1.08) and a p-value of 0.767, indicating no significant association. However, a significant effect was observed on abnormalities in coagulation test results, with an OR of 0.813 (95% CI: 0.68; 0.96) and a p-value of 0.019. In our study, we observed no correlation between the severity of drug interactions involving EGb and prescription drugs and the occurrence of bleeding, with an OR of 1.01 (95% CI: 0.94; 1.08), indicating no significant association (p = 0.767). However, this variable demonstrated a statistically significant effect on abnormalities in coagulation test results, with an OR of 0.813 (95% CI: 0.68; 0.96) and a p-value of 0.019. This suggests that although the severity of drug interactions may not influence the risk of bleeding, it does have a notable impact on the abnormalities observed in the coagulation test results.

Formatted: Justified, Line spacing: single

Formatted: Font: Font color: Black

When examining the relationship between drug interactions involving EGb and specific medications and the occurrence of bleeding, our analysis revealed that several drugs exhibited statistical significance with a p-value of less than 0.05. These drugs include clopidogrel, aspirin, celecoxib, loxoprofen, nifedipine, and omeprazole.

TheOur study identified statistically significant correlations between drug interactions involving EGb and specific medications, including clopidogrel, aspirin, celecoxib, loxoprofen, nifedipine, and omeprazole, and both the occurrence of bleeding and abnormalities in coagulation tests ($p < 0.05$). Additionally, interactions of EGb with ticagrelor, etoricoxib, insulin, omeprazole, and

domperidone were found to be associated with abnormalities in coagulation tests without affecting physician-reported bleeding.

Table 3: Association of EGb drug interactions with bleeding and abnormal coagulation tests (n=747)

	**The occurrence of bleeding**		**Abnormal coagulant test**	
Variables	OR (CI 5;95%)	P-value	OR (CI 5;95%)	P-value
Weight	1 (0.99;1)	0.607	1 (0.99;1)	0.12
Sex (Ref: Male)	1 (0.97;1.03)	0.733	1.07 (0.99;1.15)	0.071
Age (years)	1 (0.99;1)	0.325	10024 (1;1004)	0.029
BMI (mean±SD)	1 (0.99;1)	0.285	1 (0.99;1.02)	0.198
Occurrence of bleeding (Ref: yes)			1.75 (1.48;2.08)	<0.001
APTT (s)	1 (0.99;1)	0.084	1.03 (1.03;1.04)	<0.001
PT (%)	1.0009 (1.0004;1.001)	0.0005	1005 (1004;1007)	<0.001
Fibrinogen (g/l)	1.04 (1.02;1.05)	<0.001	1.22 (1190;1.25)	<0.001
Abnormal coagulant test (Ref: yes)	1.09 (1.06;1.12)	<0.001		
DDI (Ref: Yes)	1.08 (1.04;1.11)	<0.001	1.49 (1.38;1.58)	<0.001
Number of DDIs	1.06 (1.04;1.09)	<0.001	1.36 (1.3;1.42)	<0.001
Severity of DDIs (Ref: Moderate)	1.01 (0.94;1.08)	0.767	0.813 (0.68;0.96)	0.019
Clopidogrel	1.1 (1.05;1.15)	<0.001	1.58 (1.4;1.77)	<0.001
Aspirin	1.12 (1.07;1.18)	<0.001	1.48 (132;1.66)	<0.001
Ticagrelor	0.96 (0.73;1.27)	0.769	1.83 (0.92;3.61)	0.081
DOAC (enoxaban/rivaroxaban)	1.01 (0.92;1.11)	0.763	1.11 (0.88;1.39)	0.363
Acenocoumarol	0.96 (0.86;1.07)	0.45	1.07 (0.81;1.39)	0.628
Etoricoxib	1 (0.92;1.1)	0.847	1.37 (1.1;1.69)	0.0048
Celecoxib	1.11 (0.99;1.23)	0.055	0.48 (1.15;0.92)	0.0026

Meloxicam	1.06 (0.94;1.2)	0.351	1.23 (0.91;1.67)	0.185
Loxoprofen	1.58 (1.2;2.08)	0.00111	1.83 (0.93;3.61)	0.0807
Nifedipine	1.09 (1;1.18)	0.0371	1.16 (0.95;1.42)	0.139
Insulin	1 (0.92;1.09)	0.925	1.34 (1.09;1.65)	0.0054
Omeprazole	1.10 (1.04;1.15)	0.0003	1.7 (1.51;1.92)	<0.001
Domperidone	1.01 (0.92;1.12)	0.717	1.29 (1.02;1.63)	0.0325

OR: Odds Ratio; CI: Confidence Interval; BMI: Body Mass Index; SD: Standard Deviation; APTT: Activated Partial Thromboplastin Time; PT: Prothrombin Time; DDIs: Drug-Drug Interactions

Discussion

Our findings provide criticalvaluable insights into the prevalence, severity, and clinical implications of drug interactions involving EGb in conjunction withand prescription medications. We observed a considerable frequency of drug interactions, with 12.94% of the prescriptions exhibiting interactions with EGgb, which underscores the relevance of monitoring these interactions in clinical practice. A study evaluating dietary supplement use among 458 veteran outpatients receiving prescription medications found that 197 patients (43%) were using at least one dietary supplement concurrently. Of these, 89 patients (45%) had the potential for drug-dietary supplement interactions. Importantly, most of these interactions (84 or 94%) were classified as non-serious, leading to a prevalence of 6% for potentially severe interactions among those affected, and 3% (5 out of 197) among all patients taking dietary supplements alongside their medications (18). A retrospective analysis utilizing data from the Taiwan National Health Insurance Research Database spanning from 2000 to 2008 aimed to investigate the potential risk of hemorrhage associated with the concurrent prescription of EGb alongside antiplatelet or

Formatted: Justified, Line spacing: single

Formatted: Font: (Default) Times New Roman, 12 pt

Formatted: Font: (Default) Times New Roman

Formatted: Font: (Default) Times New Roman

anticoagulant agents. The findings revealed a gradual increase in the total number of prescriptions for EGb alone or in combination with antiplatelet or anticoagulant agents (21).

Among the 195 consecutive patients surveyed in this study, EGb was the predominant herbal medicine. Within the identified herb-drug interactions, eight cases involved interactions between EGb and aspirin (acetylsalicylic acid), whereas one case involved interactions between EGb and trazodone (19). These interactions were predominantly observed with drugs classified as having antiplatelet properties, anticoagulants, and NSAIDs, highlighting a critical area for vigilance among healthcare providersunderscoring the importance of recognizing potential interactions in clinical practice.

Interactions with antiplatelet drugs exhibited the highest prevalence rate and average severity level, with 69 prescriptions for clopidogrel and aspirin documented, reflecting a rate of 2.61%.

In line with our study, the prevalence of concurrent use of EGb with antiplatelet drugs among older adults varies between 5.3% and 88.3% (20). While some case reports indicate potential bleeding risks when combining *G. biloba* with anticoagulants or antiplatelet drugs, controlled clinical studies have not consistently shown significant adverse effects on hemostasis or an increased risk of bleeding (21, 22). Nonetheless, *G. biloba* may enhance the effect of cilostazol on prolonging bleeding time (23), and combining ticlopidine with *G. biloba* extract has been observed to produce greater antiplatelet effects than clopidogrel alone (24). Despite these observations, overall evidence for clinically significant interactions between *G. biloba* and antiplatelet drugs remains limited (25), highlighting the need for healthcare professionals to be aware of potential interactions and closely monitor patients taking *G. biloba* with antiplatelet medications, especially those at risk of bleeding (26).

Formatted: Font: (Default) Times New Roman, 12 pt

Formatted: Font: (Default) Times New Roman

Formatted: Vietnamese

Formatted: Font: (Default) Times New Roman

Formatted: Justified

Formatted: Font: (Default) Times New Roman

Formatted: Font: (Default) Times New Roman

Formatted: Font: (Default) Times New Roman

Formatted: Font: (Default) Times New Roman

Formatted: Font: (Default) Times New Roman

Formatted: Font: (Default) Times New Roman

Formatted: Font: (Default) Times New Roman

Formatted: Font: (Default) Times New Roman

Formatted: Font: (Default) Times New Roman

Formatted: Font: (Default) Times New Roman

Formatted: Font: (Default) Times New Roman

~~Of particular concern were interactions with antiplatelet drugs such as clopidogrel and aspirin, which manifested a notable prevalence rate and were associated with both bleeding events and abnormalities in coagulation tests.~~ Chan et al. identified an association between the risk of hemorrhage and specific patient demographics related to concurrent use of EGb and antiplatelet or anticoagulant agents, particularly patients aged ≥65 and male patients, who were prescribed EGb alone (adjusted odds ratio: 3.8 and 1.4; 95% confidence interval, 2.8–5.2 and 1.1–1.9) (27). Anticoagulant and antiplatelet therapies have been linked to a higher risk of chronic subdural hematoma in the elderly (28, 29). Nonetheless, our investigation did not reveal a correlation between age and risk of bleeding. This lack of an association may stem from the fact that the average age of the patients in our study was 60 years, revealing no discernible differences across age groups. The primary interaction observed was between *G. biloba*~~Ginkgo~~ and aspirin, which has several significant implications. First, both substances are commonly used by older adults (30). Second, they are readily available as over-the-counter medications, allowing patients to acquire them without the oversight of healthcare professionals. Older individuals often seek *G. biloba*~~Ginkgo~~ to enhance memory, whereas aspirin is frequently prescribed to manage cardiovascular conditions. There is documented evidence indicating that concurrent use of these substances may increase the risk of bleeding associated with aspirin. The adverse outcomes stemming from this interaction can be severe. A case of spontaneous bleeding linked to the simultaneous intake of *G. biloba*~~Ginkgo~~ and aspirin has been reported (14). A cross-sectional survey evaluated the use of herbal medicines that may interfere with the effects of antiplatelet or anticoagulant therapy among 250 patients in medical wards. Overall, almost 31% (n=23, N=76) of patients were taking one or more of the specified herbal medicines (ginseng [Panax ginseng], garlic [Allium sativum], and ~~Ginkgo [~~*G. biloba*~~]~~) thought to interact with antiplatelet or

anticoagulant therapy. The study showed that 21% (n=16, N=76) of patients co-ingested specific herbs with antiplatelet or anticoagulant therapy, of which half were at risk of potential drug–herb interactions (31).

We did not observe a correlation between the interactions involving *G. biloba* and acenocoumarol. While some studies suggest minimal risk, others indicate potential concerns. In vitro studies showed inhibition of CYP2C9 by *G. biloba*~~Ginkgo~~ extract, but in vivo studies found no significant interactions with CYP2C9 substrates (32). controlled studies using the standardized extract EGb 761 showed no significant impact on hemostasis or adverse effects when combined with aspirin or warfarin (21). ~~In contrast to our study,~~ Stoddard and colleagues employed text-processing techniques to extract data on *G. biloba*~~Ginkgo~~ usage and integrated it with prescription records for warfarin from a large clinical database. Their statistical analysis indicated that the concurrent use of *G. biloba*~~Ginkgo~~ with warfarin significantly increased the risk of adverse bleeding events in patients (hazard ratio = 1.38, 95% CI: 1.20 to 1.58, $p < 0.001$). Disregarding time-at-risk, a bleeding event occurred in 210 (3.1%) patients in the warfarin plus *G. biloba*~~Ginkgo~~ group compared to 38,408 (5.4%) patients in the warfarin-only group (15). ~~An investigation was conducted in mice to assess the potential of EGb to induce spontaneous bleeding or enhance the anticoagulant effects of warfarin through herb-drug interactions. The findings revealed that EGb and ginkgolide B did not affect blood coagulation *in vivo*. Interestingly, EGb mitigated the anticoagulant effects of warfarin by inducing hepatic cytochrome P450 enzymes via bilobalide (35).~~

Interestingly, interactions with medications such as omeprazole, while exhibiting a relatively high frequency, were classified as mild. Eighteen healthy Chinese participants were recruited to explore the potential herb-drug interactions between EGb and omeprazole, a commonly

prescribed CYP2C19 substrate, across different CYP2C19 genotypes. The findings from this study indicate that EGb can induce omeprazole hydroxylation, with the extent of induction varying based on the individual's CYP2C19 genotype. Additionally, the concurrent administration of EGb decreased the renal clearance of 5-hydroxyomeprazole. These results suggest that coadministration of EGb with omeprazole or other CYP2C19 substrates may significantly affect their efficacy (33).

Nonetheless, these interactions still warrant attention owing to their potential impact on coagulation profiles. Further analysis revealed a significant association between drug interactions involving *G. biloba* and the occurrence of bleeding and coagulation test abnormalities. Clinical studies have shown that *G. biloba* does not affect the APTT, PT, or fibrinogen indices (34). However, 15 case reports published in the literature have delineated the temporal correlation between *G. biloba* administration and the occurrence of bleeding events (12). Most of these cases involved severe medical conditions, with eight incidents specifically involving intracranial bleeding. Nevertheless, other risk factors for bleeding were identified among the 13 case reports. Six reports documented the cessation of *G. biloba* intake, which resulted in the absence of recurrent bleeding episodes. Additionally, three reports indicated elevated bleeding times in patients with *G. biloba* intake (12).

Our findings underscore the need for clinicians to be vigilant in assessing potential interactions, particularly when prescribing *G. biloba* in conjunction with medications known to increase bleeding risk. Additionally, the study revealed no correlation between the severity of drug interactions involving EGb and the incidence of bleeding, as indicated by an odds ratio (OR) of 1.01 (95% CI: 0.94; 1.08) and a p-value of 0.767, demonstrating no significant association. Conversely, a significant effect was noted regarding abnormalities in coagulation test results,

Formatted: Justified

Formatted: Font: (Default) Times New Roman

with an OR of 0.813 (95% CI: 0.68; 0.96) and a p-value of 0.019~~although the severity of drug interactions did not significantly influence the risk of bleeding, it had a notable impact on the abnormalities observed in the coagulation test results~~. Research on the interactions between *G. biloba* and anticoagulant and antiplatelet drugs presents mixed findings. Potential mechanisms of interaction may include pharmacodynamic effects or the induction of hepatic cytochrome P450 enzymes (35). The severity of these interactions varies from moderate to severe (26). While some cases report an increased risk of bleeding, pharmacokinetic studies show inconsistent results (36). This emphasizes the importance of considering the severity of the interactions between clinical decision-making and patient management. The observed associations between specific medications and the occurrence of bleeding and coagulation abnormalities further highlight the need for careful evaluation and monitoring of patients receiving *G. biloba* therapy. Clopidogrel, aspirin, celecoxib, loxoprofen, nifedipine, and omeprazole were among the medications significantly associated with these outcomes, warranting particular attention in clinical practice.

Our study had some limitations. First, the study sample was derived from patients who were prescribed *G. biloba*; this may have introduced selection bias. Patients who choose to use or are prescribed *G. biloba* may possess different characteristics compared to those who do not use the supplement, potentially impacting the generalizability of our findings. Second, despite our efforts to collect comprehensive medical records, some data, particularly laboratory tests, including APTT, PT, and fibrinogen levels, may have been missing or incomplete. This incompleteness could compromise the accuracy and reliability of our results, leading to a potential underestimation or overestimation of the prevalence of drug interactions and bleeding risk. Additionally, our study may not account for all potential confounding variables that could

Formatted: Font: (Default) Times New Roman
Formatted: Font: (Default) Times New Roman, Italic
Formatted: Font: Italic
Formatted: Font: (Default) Times New Roman, Italic
Formatted: Font: (Default) Times New Roman
Formatted: Font: (Default) Times New Roman
Formatted: Font: (Default) Times New Roman
Formatted: Font: (Default) Times New Roman

influence the bleeding risk and abnormal coagulation profiles. Factors such as comorbidities, concurrent medications, lifestyle habits, and dietary patterns, which may have influenced the associations observed in our study, were not thoroughly assessed. These limitations should be considered when interpreting the results of this study.

Conclusion

In conclusion, this study provides valuable insights into the prevalence and implications of drug interactions involving EGb on bleeding risk and coagulation profiles. A significant prevalence of drug interactions was observed, primarily with antiplatelets, anticoagulants, and NSAIDs. Notably, interactions with anticoagulants such as direct oral anticoagulants (DOACs) and acenocoumarol were not significant in this study. Among these interactions, antiplatelet medications like clopidogrel and aspirin exhibited the highest prevalence, while omeprazole was frequently involved, though with mild severity. The findings revealed a strong association between clinical bleeding and abnormal coagulation, highlighting notable correlations between EGb drug interactions, bleeding risk, and coagulation abnormalities. Interestingly, while the severity of EGb drug interactions did not correlate with bleeding risk, it did impact coagulation test results. Specific medications, including clopidogrel, aspirin, celecoxib, loxoprofen, nifedipine, and omeprazole, were significantly linked to both bleeding risk and abnormal coagulation~~this retrospective observational study provides valuable insights into the prevalence and implications of drug interactions involving EGb on bleeding risk and coagulation profiles. We found a notable prevalence of drug interactions, primarily involving antiplatelets, anticoagulants, and NSAIDs. Interestingly, interactions with anticoagulants, such as DOACs and~~

Formatted: Justified

Formatted: Font: (Default) Times New Roman

acenocoumarol, were not significant in our analysis. Among these interactions, antiplatelet drugs such as clopidogrel and aspirin exhibited the highest prevalence. Omeprazole also emerged as a frequently interacting drug, albeit with mild severity. Our analysis revealed a strong association between clinical bleeding and abnormal coagulation results, as well as significant correlations between EGb drug interactions, bleeding risk, and abnormal coagulation. Notably, the severity of EGb drug interactions did not correlate with bleeding risk but did influence abnormal coagulation test results. Specific medications, including clopidogrel, aspirin, celecoxib, loxoprofen, nifedipine, and omeprazole, were significantly correlated with bleeding risk and abnormal coagulation. Overall, our findings underscore the importance of considering EGb drug interactions in bleeding risk assessments and coagulation management, highlighting the need for further research in this area to optimize patient safety and outcomes.

Acknowledgments

We would like to express our gratitude to all the physicians and nurses in the Outpatient Clinic and Laboratory Department of Hai Phong International Hospital for their assistance in patient recruitment and clinical laboratory testing for our study. Furthermore, we greatly appreciate the invaluable support provided by Hai Phong University of Medicine and Pharmacy for our Biomedical - Pharmaceutical Sciences Research Group in this endeavor.

References

1. McKenna DJ, Jones K, Hughes K. Efficacy, safety, and use of ginkgo biloba in clinical and preclinical applications. Altern Ther Health Med. 2001;7(5):70-86, 8-90.
2. Singh SK, Srivastav S, Castellani RJ, Plascencia-Villa G, Perry G. Neuroprotective and Antioxidant Effect of Ginkgo biloba Extract Against AD and Other Neurological Disorders. Neurotherapeutics. 2019;16(3):666-74.
3. Ginkgo. Drugs and Lactation Database (LactMed(R)). Bethesda (MD)2006.
4. Tchantchou F, Xu Y, Wu Y, Christen Y, Luo Y. EGb 761 enhances adult hippocampal neurogenesis and phosphorylation of CREB in transgenic mouse model of Alzheimer's disease. FASEB J. 2007;21(10):2400-8.
5. Yoshitake T, Yoshitake S, Kehr J. The Ginkgo biloba extract EGb 761(R) and its main constituent flavonoids and ginkgolides increase extracellular dopamine levels in the rat prefrontal cortex. Br J Pharmacol. 2010;159(3):659-68.
6. Marcilhac A, Dakine N, Bourhim N, Guillaume V, Grino M, Drieu K, et al. Effect of chronic administration of Ginkgo biloba extract or Ginkgolide on the hypothalamic-pituitary-adrenal axis in the rat. Life Sci. 1998;62(25):2329-40.
7. Le Bars PL, Katz MM, Berman N, Itil TM, Freedman AM, Schatzberg AF. A placebo-controlled, double-blind, randomized trial of an extract of Ginkgo biloba for dementia. North American EGb Study Group. JAMA. 1997;278(16):1327-32.
8. Birks J, Grimley Evans J. Ginkgo biloba for cognitive impairment and dementia. Cochrane Database Syst Rev. 2009(1):CD003120.

9. Tan MS, Yu JT, Tan CC, Wang HF, Meng XF, Wang C, et al. Efficacy and adverse effects of ginkgo biloba for cognitive impairment and dementia: a systematic review and meta-analysis. J Alzheimers Dis. 2015;43(2):589-603.

10. Unger M. Pharmacokinetic drug interactions involving Ginkgo biloba. Drug Metab Rev. 2013;45(3):353-85.

11. Kellermann AJ, Kloft C. Is there a risk of bleeding associated with standardized Ginkgo biloba extract therapy? A systematic review and meta-analysis. Pharmacotherapy. 2011;31(5):490-502.

12. Bent S, Goldberg H, Padula A, Avins AL. Spontaneous bleeding associated with ginkgo biloba: a case report and systematic review of the literature: a case report and systematic review of the literature. J Gen Intern Med. 2005;20(7):657-61.

13. Ang-Lee MK, Moss J, Yuan CS. Herbal medicines and perioperative care. JAMA. 2001;286(2):208-16.

14. Rosenblatt M, Mindel J. Spontaneous hyphema associated with ingestion of Ginkgo biloba extract. N Engl J Med. 1997;336(15):1108.

15. Stoddard GJ, Archer M, Shane-McWhorter L, Bray BE, Redd DF, Proulx J, et al. Ginkgo and Warfarin Interaction in a Large Veterans Administration Population. AMIA Annu Symp Proc. 2015;2015:1174-83.

16. Kohler S, Funk P, Kieser M. Influence of a 7-day treatment with Ginkgo biloba special extract EGb 761 on bleeding time and coagulation: a randomized, placebo-controlled, double-blind study in healthy volunteers. Blood Coagul Fibrinolysis. 2004;15(4):303-9.

17. R: TR. A language and environment for statistical computing. R Foundation for Statistical Computing. 2005.

18. Peng CC, Glassman PA, Trilli LE, Hayes-Hunter J, Good CB. Incidence and severity of potential drug-dietary supplement interactions in primary care patients: an exploratory study of 2 outpatient practices. Arch Intern Med. 2004;164(6):630-6.

19. Dergal JM, Gold JL, Laxer DA, Lee MS, Binns MA, Lanctot KL, et al. Potential interactions between herbal medicines and conventional drug therapies used by older adults attending a memory clinic. Drugs Aging. 2002;19(11):879-86.

20. Agbabiaka TB, Wider B, Watson LK, Goodman C. Concurrent Use of Prescription Drugs and Herbal Medicinal Products in Older Adults: A Systematic Review. Drugs Aging. 2017;34(12):891-905.

21. Bone KM. Potential interaction of Ginkgo biloba leaf with antiplatelet or anticoagulant drugs: what is the evidence? Mol Nutr Food Res. 2008;52(7):764-71.

22. Gaby AR, editor Ginkgo Biloba Extract: A Review1996.

23. Aruna D, Naidu MU. Pharmacodynamic interaction studies of Ginkgo biloba with cilostazol and clopidogrel in healthy human subjects. Br J Clin Pharmacol. 2007;63(3):333-8.

24. Hong JM, Shin DH, Lim YA, Lee JS, Joo IS. Ticlopidine with Ginkgo Biloba extract: a feasible combination for patients with acute cerebral ischemia. Thromb Res. 2013;131(4):e147-53.

25. Abad MJ, Bedoya LM, Bermejo P. An update on drug interactions with the herbal medicine Ginkgo biloba. Curr Drug Metab. 2010;11(2):171-81.

26. Tsai HH, Lin HW, Lu YH, Chen YL, Mahady GB. A review of potential harmful interactions between anticoagulant/antiplatelet agents and Chinese herbal medicines. PLoS One. 2013;8(5):e64255.

27. Chan AL, Leung HW, Wu JW, Chien TW. Risk of hemorrhage associated with co-prescriptions for Ginkgo biloba and antiplatelet or anticoagulant drugs. J Altern Complement Med. 2011;17(6):513-7.

28. De Bonis P, Trevisi G, de Waure C, Sferrazza A, Volpe M, Pompucci A, et al. Antiplatelet/anticoagulant agents and chronic subdural hematoma in the elderly. PLoS One. 2013;8(7):e68732.

29. Nathan S, Goodarzi Z, Jette N, Gallagher C, Holroyd-Leduc J. Anticoagulant and antiplatelet use in seniors with chronic subdural hematoma: Systematic review. Neurology. 2017;88(20):1889-93.

30. Gold JL, Laxer DA, Dergal JM, Lanctot KL, Rochon PA. Herbal-drug therapy interactions: a focus on dementia. Curr Opin Clin Nutr Metab Care. 2001;4(1):29-34.

31. Saw JT, Bahari MB, Ang HH, Lim YH. Potential drug-herb interaction with antiplatelet/anticoagulant drugs. Complement Ther Clin Pract. 2006;12(4):236-41.

32. Mohutsky MA, Anderson GD, Miller JW, Elmer GW. Ginkgo biloba: evaluation of CYP2C9 drug interactions in vitro and in vivo. Am J Ther. 2006;13(1):24-31.

33. Yin OQ, Tomlinson B, Waye MM, Chow AH, Chow MS. Pharmacogenetics and herb-drug interactions: experience with Ginkgo biloba and omeprazole. Pharmacogenetics. 2004;14(12):841-50.

34. Bal Dit Sollier C, Caplain H, Drouet L. No alteration in platelet function or coagulation induced by EGb761 in a controlled study. Clin Lab Haematol. 2003;25(4):251-3.

35. Taki Y, Yokotani K, Yamada S, Shinozuka K, Kubota Y, Watanabe Y, et al. Ginkgo biloba extract attenuates warfarin-mediated anticoagulation through induction of hepatic cytochrome P450 enzymes by bilobalide in mice. Phytomedicine. 2012;19(2):177-82.

36. Li J, Liang Q, Sun G. Interaction between Traditional Chinese Medicine and Anticoagulant/Antiplatelet Drugs. Curr Drug Metab. 2019;20(9):701-13.

Response to reviewers:

The authors did well in the conceptualization of the study. However, the manuscript was not properly written. The result and discussion sessions need a major revision. There was no Figure 1 in the result session. I have highlighted areas where revision should be made.

Thank you for your feedback regarding our manuscript. We appreciate your recognition of the conceptualization of the study.

We acknowledge your comments about the need for major revisions in the results and discussion sections; we have taken the necessary steps to enhance clarity and coherence throughout these areas.

Regarding Figure 1, we understand that it was submitted separately in accordance with the guidelines of PLOS ONE. However, we will resubmit Figure 1 along with the revised manuscript to ensure it is readily available for review and aligns with the manuscript content.

We believe that the revisions made have significantly improved the manuscript, and we appreciate your valuable guidance in this process. Thank you for your consideration.

Line 23-40:

1. start the abstract with a little introduction on Gingo biloba

2. there is no methodology in this abstract. What was the selection criteria?

kindly include statistical analysis that was used.

Thank you for your insightful feedback regarding our abstract. We have made the recommended changes to enhance its clarity and comprehensiveness.

1. We have added an introductory statement about Ginkgo biloba at the beginning of the abstract to provide context for the study. This introduction helps establish the significance of Ginkgo biloba in relation to the research conducted.
2. We acknowledge the lack of methodological details in the original abstract. In response to your comments, we have included the selection criteria used for participant inclusion in the study. Additionally, we have specified the statistical analysis methods employed to assess the data.

The following paragraph has been added to our abstract to address these points:

"Our analysis utilized data from patients admitted to Hai Phong International Hospital between January 2022 and December 2023. Inclusion criteria consisted of patients aged 18 years and above, those prescribed *Ginkgo biloba* extract alone or in combination with other medications, and the availability of complete medical records, including medication history, laboratory tests, and clinical outcomes"

3. Add keywords

The following keywaords has been added to our manuscript:

Ginkgo biloba; drug interactions; bleeding risk; anticoagulants; coagulation profiles

Line 47 -48: reference???

In response to your inquiry about the reference for lines 47-48, we have added the appropriate citation to support the statements made in that section.

Line 51: repetition: *G. biloba* leaf extract (EGb) is widely recognized as one of the most popular health supplements globally due to its benefits for mental focus. EGb 761, the standardized EGb, is commonly used in studies investigating its effects. Research has indicated that EGb influences several neurotransmitter pathways and brain structures, particularly those observed in animal studies

In response to your feedback, we have revised this section to eliminate redundant phrases.

Line 73: Even though EGb is commonly used, it has risks, especially in terms of its potential interactions with prescription medications. These interactions can lead to issues like bleeding disorders and abnormal coagulation profiles. Consequently, caution is advised when administering EGb to patients with bleeding disorders or those who are taking NSAIDs, antiplatelet drugs, or anticoagulants.

In response to your feedback, we have revised this section based on your suggestions

Line 81: Recently, several case reports have suggested a possible connection between hemorrhagic complications and the use of EGb preparations. In response, a trial was carried out to evaluate the effects of the Ginkgo biloba special extract, EGb 761, on hemostatic parameters. The study found that, out of the 29 coagulation and bleeding parameters examined, EGb 761 did not show any evidence of inhibiting blood coagulation or platelet aggregation.

In response to your feedback, we have revised this section based on your suggestions

Line 114: Study design

In response to your feedback, we have revised this section based on your suggestions as following:

– Patients aged 18 years and older

– Patients prescribed EGb either alone or alongside other prescription medications

– Availability of complete medical records, including medication history, laboratory tests, and clinical outcomes.

This criterion was specifically applied to examine the impact of EGb interactions on bleeding occurrences or abnormal coagulation tests.

Line 176-194: kindly summarize. some of the information should be moved to the discussion session

Discussion was poorly written.

Kindly rewrite this section.

1. the result was not properly discussed. kindly discuss the result . move some of the information in the results section to this section

2. authors should check for grammar. use the right tenses.

Thank you for your constructive feedback regarding the discussion section of our manuscript. We appreciate your attention to detail and the suggestions for improvement.

In response to your comments, we have rewritten the discussion to provide a more thorough analysis of the results. We have moved relevant information from the results section to ensure that findings are properly contextualized and discussed in detail.

Additionally, we have carefully reviewed the discussion for grammatical accuracy and proper use of tenses, making necessary adjustments to enhance clarity and readability.

We believe that the revisions have significantly strengthened the discussion section, and we appreciate your guidance in helping us improve the overall quality of the manuscript. Thank you for your valuable input.
