# Supplementary material for: Impact of Ginkgo biloba drug interactions on bleeding risk and coagulation profiles: A comprehensive analysis
Source: PLoS One. 2025 Apr 8;20(4):e0321804. doi: 10.1371/journal.pone.0321804 (PMC11991284; doi:10.1371/journal.pone.0321804)
Supplement: Attachment — Submitted filename: Response to reviewers.docx [file pone.0321804.s006.docx]

**Response to reviewers:**

The authors did well in the conceptualization of the study. However, the manuscript was not properly written. The result and discussion sessions need a major revision. There was no Figure 1 in the result session. I have highlighted areas where revision should be made.

Thank you for your feedback regarding our manuscript. We appreciate your recognition of the conceptualization of the study.

We acknowledge your comments about the need for major revisions in the results and discussion sections; we have taken the necessary steps to enhance clarity and coherence throughout these areas.

Regarding Figure 1, we understand that it was submitted separately in accordance with the guidelines of PLOS ONE. However, we will resubmit Figure 1 along with the revised manuscript to ensure it is readily available for review and aligns with the manuscript content.

We believe that the revisions made have significantly improved the manuscript, and we appreciate your valuable guidance in this process. Thank you for your consideration.

**Line 23-40:**

1. start the abstract with a little introduction on Gingo biloba

2. there is no methodology in this abstract. What was the selection criteria?

kindly include statistical analysis that was used.

Thank you for your insightful feedback regarding our abstract. We have made the recommended changes to enhance its clarity and comprehensiveness.

1. We have added an introductory statement about Ginkgo biloba at the beginning of the abstract to provide context for the study. This introduction helps establish the significance of Ginkgo biloba in relation to the research conducted.
2. We acknowledge the lack of methodological details in the original abstract. In response to your comments, we have included the selection criteria used for participant inclusion in the study. Additionally, we have specified the statistical analysis methods employed to assess the data.

The following paragraph has been added to our abstract to address these points:

“Our analysis utilized data from patients admitted to Hai Phong International Hospital between January 2022 and December 2023. Inclusion criteria consisted of patients aged 18 years and above, those prescribed *Ginkgo biloba* extract alone or in combination with other medications, and the availability of complete medical records, including medication history, laboratory tests, and clinical outcomes”

3. Add keywords

The following keywaords has been added to our manuscript:

**Ginkgo biloba; drug interactions; bleeding risk; anticoagulants;** **coagulation profiles**

**Line 47 -48: reference???**

In response to your inquiry about the reference for lines 47-48, we have added the appropriate citation to support the statements made in that section.

**Line 51:** repetition: *G. biloba* leaf extract (EGb) is widely recognized as one of the most popular health supplements globally due to its benefits for mental focus. EGb 761, the standardized EGb, is commonly used in studies investigating its effects. Research has indicated that EGb influences several neurotransmitter pathways and brain structures, particularly those observed in animal studies

In response to your feedback, we have revised this section to eliminate redundant phrases.

**Line 73:** Even though EGb is commonly used, it has risks, especially in terms of its potential interactions with prescription medications. These interactions can lead to issues like bleeding disorders and abnormal coagulation profiles. Consequently, caution is advised when administering EGb to patients with bleeding disorders or those who are taking NSAIDs, antiplatelet drugs, or anticoagulants.

In response to your feedback, we have revised this section based on your suggestions

**Line 81:** Recently, several case reports have suggested a possible connection between hemorrhagic complications and the use of EGb preparations. In response, a trial was carried out to evaluate the effects of the Ginkgo biloba special extract, EGb 761, on hemostatic parameters. The study found that, out of the 29 coagulation and bleeding parameters examined, EGb 761 did not show any evidence of inhibiting blood coagulation or platelet aggregation.

In response to your feedback, we have revised this section based on your suggestions

**Line 114:** Study design

In response to your feedback, we have revised this section based on your suggestions as following:

- Patients aged 18 years and older
- Patients prescribed EGb either alone or alongside other prescription medications
- Availability of complete medical records, including medication history, laboratory tests, and clinical outcomes.

This criterion was specifically applied to examine the impact of EGb interactions on bleeding occurrences or abnormal coagulation tests.

**Line 176-194:** kindly summarize. some of the information should be moved to the discussion session

Discussion was poorly written.

Kindly rewrite this section.

1. the result was not properly discussed. kindly discuss the result . move some of the information in the results section to this section

2. authors should check for grammar. use the right tenses.

Thank you for your constructive feedback regarding the discussion section of our manuscript. We appreciate your attention to detail and the suggestions for improvement.

In response to your comments, we have rewritten the discussion to provide a more thorough analysis of the results. We have moved relevant information from the results section to ensure that findings are properly contextualized and discussed in detail.

Additionally, we have carefully reviewed the discussion for grammatical accuracy and proper use of tenses, making necessary adjustments to enhance clarity and readability.

We believe that the revisions have significantly strengthened the discussion section, and we appreciate your guidance in helping us improve the overall quality of the manuscript. Thank you for your valuable input.
