# Supplementary material for: Impact of Ginkgo biloba drug interactions on bleeding risk and coagulation profiles: A comprehensive analysis
Source: PLoS One. 2025 Apr 8;20(4):e0321804. doi: 10.1371/journal.pone.0321804 (PMC11991284; doi:10.1371/journal.pone.0321804)
Supplement: Attachment — Submitted filename: PONE-D-24-33374_R1_reviewer.docx [file pone.0321804.s004.docx]

The authors responded to most of the comments.
authors did NOT respond satisfactory to the following main points:

“Discussion was poorly written. Kindly rewrite this section. 1. the result was not properly discussed. kindly discuss the result . move some of the information in the results section to this section 2. authors should check for grammar. use the right tenses.”

Results were not adequately discussed.
