# Supplementary material for: Impact of Ginkgo biloba drug interactions on bleeding risk and coagulation profiles: A comprehensive analysis
Source: PLoS One. 2025 Apr 8;20(4):e0321804. doi: 10.1371/journal.pone.0321804 (PMC11991284; doi:10.1371/journal.pone.0321804)
Supplement: Attachment — Submitted filename: Response_to_Reviewers_auresp_2.docx [file pone.0321804.s007.docx]

**Response to Reviewers**

Manuscript ID: PONE-D-24-33374R1
Title: *Impact of Ginkgo biloba drug interactions on bleeding risk and coagulation profiles: A comprehensive analysis*

Dear Dr. Ogunlakin and Reviewers,

We would like to thank you for the opportunity to revise our manuscript and for the constructive feedback provided by the reviewers. We have carefully considered all the comments and have revised the manuscript accordingly. Below, we provide a detailed response to each comment, including how we have addressed them in the revised manuscript.

**Response to Reviewer #2**

**Comment 1:** *The authors have not addressed the comments raised in the previous review. I'll advise them to go through the previous reviewed document and address the comments, especially the results and discussion section. I have also highlighted some areas that need to be revised.*

**Response**:
We regret that the previous revision did not fully address the concerns raised. In this revision, we have comprehensively reviewed the previous feedback and revised the manuscript accordingly, with a particular focus on the Results and Discussion sections. The following improvements have been made:

- **Results Section**:
- Key findings have been clarified and presented in a more structured format to enhance readability and alignment with the Discussion section.
- Statistical findings and their clinical relevance have been highlighted to provide a clear understanding of their implications.
- **Discussion Section**:
- The section has been rewritten to provide a detailed interpretation of the results in the context of existing literature.
- Relevant information from the Results section has been relocated to the Discussion where appropriate to ensure logical flow and avoid redundancy.
- **Grammar and Clarity**:
- The manuscript has been thoroughly revised for grammatical accuracy, proper tense usage, and improved readability.

**Response to Reviewer #3**

**Comment 1:** *Discussion was poorly written. Kindly rewrite this section. The result was not properly discussed. Kindly discuss the result. Move some of the information in the Results section to this section.*

**Response:**
We have completely rewritten the Discussion section to address this concern. Specific revisions include:

- A detailed analysis of key findings, including the prevalence and severity of drug interactions, correlations with clinical bleeding and coagulation test abnormalities, and implications for clinical practice.
- Integration of relevant literature to contextualize the findings and highlight their significance.
- Relocation of interpretative insights from the Results section to the Discussion to enhance logical flow and depth of analysis.

**Comment 2:** *Authors should check for grammar. Use the right tenses.*

**Response:** The manuscript has been thoroughly reviewed and revised for grammar, tense consistency, and overall readability.

**General revisions across the manuscript**

1. **Statistical Analysis:** We have double-checked the statistical methodologies and included clarifications where needed to ensure rigor.
2. **Figures and Tables:** All figures and tables have been reviewed for clarity and alignment with the text. We used the PACE digital diagnostic tool to confirm compliance with PLOS requirements.
3. **Data Availability:** We have reconfirmed the data availability statement and ensured that all data are accessible as described.

We believe these revisions address the reviewers’ and editor’s concerns and significantly enhance the quality of our manuscript. The revised manuscript with tracked changes and the clean version have been uploaded as separate files.

We appreciate your consideration and look forward to your feedback.

Sincerely,
